# BIN1 inhibited tumor growth, metastasis and stemness by ALDH1/NOTCH pathway in bladder carcinoma

**DOI:** 10.1186/s41065-025-00384-w

**Published:** 2025-02-27

**Authors:** Si-yu Chen, Ya-long Zhang, Xiao-ran Li, Ji-rong Wang, Kun-peng Li, Shun Wan, Jian-wei Yang, Hao Wang, Jin-long Cao, Chen-yang Wang, Xin-peng Fan, Sheng-jun Fu, Li-yun Ding, Tuan-jie Che, Li Yang

**Affiliations:** 1 Department of Urology, The Second Hospital of Lanzhou University, Lanzhou, China; 2 Gansu Province Clinical Research Center for Urinary System Disease, Lanzhou, China; 3 School of Physical Science and Technology, Lanzhou University, Lanzhou, China; 4 Baiyuan Company for Gene Technology, Lanzhou, China

**Keywords:** BIN1, Bladder cancer, Cancer stem cells, ALDH1, NOTCH, EMT

## Abstract

**Background:**

Bladder cancer (BLCA) represents one of the most prevalent urological malignancies worldwide. Bridging integrator 1 (BIN1), a well-characterized tumor suppressor that interacts with and inhibits oncogenic Myc transcription factors, has demonstrated crucial roles in various cancer types. However, its specific functions and underlying molecular mechanisms in BLCA development and progression remain poorly understood. This study aims to elucidate the role of BIN1 in regulating BLCA cell proliferation, metastasis, and cancer stem cell properties.

**Methods:**

Using urinary proteomics analysis, we identified BIN1 as a significantly dysregulated protein in BLCA. The clinical significance of BIN1 was further validated through comprehensive analyses of public databases. BIN1 expression levels defined distinct molecular and immunological subtypes of BLCA. Through proteomic profiling of BIN1-overexpressing UMUC3 cells and corresponding controls, we identified ALDH1 as a key downstream effector in the BIN1-regulated ALDH1/NOTCH signaling axis. We employed multiple experimental approaches, including Western blot analysis, quantitative RT-PCR, immunofluorescence staining, wound healing assays, transwell migration assays, colony formation assays, tumor sphere formation assays, flow cytometry, CCK8 proliferation assays, and cell transfection experiments.

**Results:**

We observed significant downregulation of BIN1 in both BLCA tissues and cell lines compared to normal adjacent tissues and SV-HUC-1 cells, respectively. BIN1 overexpression inhibited cancer cell proliferation by promoting apoptosis and suppressed epithelial-mesenchymal transition (EMT), thereby reducing local invasion and distant metastasis. Additionally, BIN1 regulated cancer stem cell properties through modulation of ALDH1 expression, with NOTCH2 acting as a crucial downstream mediator of ALDH1 signaling.

**Conclusion:**

Our findings demonstrate that BIN1 functions as a tumor suppressor in BLCA and suggest its potential utility as both a diagnostic biomarker and therapeutic target for BLCA treatment.

**Supplementary Information:**

The online version contains supplementary material available at 10.1186/s41065-025-00384-w.

## Introduction

Bladder cancer (BLCA), with an estimated annual incidence of 500,000 new cases and 200,000 fatalities worldwide, represents a significant global health challenge [1]. Urothelial carcinoma accounts for approximately 95% of all BLCA cases [2] and is classified into two major categories: non-muscle-invasive BLCA (NMIBC; stages Tis, Ta, and T1) and muscle-invasive BLCA (MIBC; stages T2-T4). These distinct categories require significantly different therapeutic strategies [3, 4]. Approximately 10–30% of NMIBC cases progress to MIBC, with potential development of metastatic disease. The overall prognosis of BLCA remains poor, with up to 50% of patients experiencing recurrence or distant metastasis [5–7].

Early detection of BLCA significantly improves patient survival rates, highlighting the critical need for developing minimally invasive diagnostic approaches. Urine-based assays, particularly urinary proteomics, have emerged as promising tools for disease diagnosis, monitoring, minimal residual disease detection, and prediction of metastatic recurrence. Understanding the underlying biological and molecular mechanisms that drive BLCA progression and metastasis is essential for developing more effective therapeutic strategies.

Cancer stem cells (CSCs) represent a crucial subpopulation of tumor cells that drive tumor metastasis and poor clinical outcomes. These cells exhibit distinct characteristics, including tumor-initiating capacity, self-renewal ability, and multi-lineage differentiation potential. The precise regulation of self-renewal and differentiation processes is essential for tumor growth and metastatic spread. Therefore, therapeutic strategies targeting CSCs have emerged as a promising approach to improve survival outcomes in patients with metastatic disease [8, 9].

Bridging integrator 1 (BIN1), also known as Amphiphysin 2 or SH3P9, is a ubiquitously expressed nucleo-cytoplasmic protein that was initially characterized as a Myc-interacting partner [10]. While BIN1 shows widespread tissue distribution, it demonstrates notably high expression levels in brain and muscle tissues [11, 12]. BIN1 undergoes extensive alternative splicing, generating multiple isoforms with diverse cellular functions, including endocytosis, membrane trafficking, cytoskeletal organization, DNA repair, and apoptosis regulation. Notably, BIN1 plays a critical role in mediating Myc-induced apoptosis in transformed cells [13]. The nuclear-localized BIN1 isoform specifically antagonizes Myc-mediated transformation and induces p53-independent apoptosis across various cancer types, including breast, prostate, melanoma, astrocytoma, and neuroblastoma. Reduced or lost BIN1 expression is frequently observed in these malignancies, supporting its role as a tumor suppressor [10, 14–20]. In addition to its Myc-dependent functions, BIN1 regulates tumor cell growth through multiple signaling pathways [15, 16]. Nevertheless, the specific role and pattern of BIN1 expression in the pathogenesis of human cancers, especially BLCA, remain largely unexplored.

Aldehyde dehydrogenases (ALDHs) constitute a family of NADH-dependent enzymes that catalyze the oxidative conversion of aldehydes to carboxylic acids. ALDH1, a key member of this family also known as retinaldehyde dehydrogenase 1 (RALHD1), primarily catalyzes the conversion of retinaldehyde to retinoic acid in vitamin A metabolism [21]. Upon synthesis, RA translocates to the nucleus where it acts as a ligand for retinoic acid receptors and retinoid X receptors. These nuclear receptors function as transcription factors, regulating genes essential for cellular differentiation, proliferation, and lipid metabolism [22]. Emerging evidence has established ALDH1 activity as a characteristic marker of both normal stem cells, particularly hematopoietic stem cells, and CSCs across multiple tissue types [23–25]. This association highlights ALDH1’s fundamental role in stem cell biology and cancer development.

In this study, we demonstrate, for the first time, a marked underexpression of BIN1 in BLCA tissues relative to normal counterparts. Furthermore, our findings indicate that BIN1 suppresses proliferation, metastasis, and the stem-like properties of BLCA cells. The specific aims of this study were to characterize BIN1’s expression pattern in BLCA, investigate its functional role in cancer progression, and decipher its regulatory mechanisms, particularly focusing on the ALDH1/NOTCH signaling pathway. Elucidating these molecular mechanisms and functions of BIN1 not only sheds light on the pathogenic progression of BLCA but also positions BIN1 as a potential novel biomarker and therapeutic target for BLCA diagnosis and treatment, particularly in metastatic cases.

## Materials and methods

### Sample data source

RNA sequencing data and associated clinical information were obtained from multiple comprehensive databases. The primary analysis utilized The Cancer Genome Atlas (TCGA) dataset, comprising 414 BLCA tissue samples and 13 normal bladder tissue controls (total *n* = 427). Clinical characteristics of BLCA cases were extracted from the GSE13507 microarray dataset available in the Gene Expression Omnibus (GEO) database. Proteomic analyses integrated data from our previously published study examining urine samples from 5 BLCA patients and 5 healthy controls [26], supplemented with additional proteomic data from Zhang et al. [27]. Analysis of epigenetically regulated mRNA expression indices (EREG-mRNAsi) was performed using TCGA-BLCA data, following methodology established in previous research [28].

### WGCNA analysis

We first analyzed differential expression between tumor and normal samples in the TCGA RNA-sequencing dataset. We applied stringent selection criteria (*p* < 0.05 and log2 fold change > 1) to identify significantly altered genes (Supplementary Table S1). After constructing a preliminary clustering tree to identify and exclude outliers, we performed Weighted Gene Co-expression Network Analysis (WGCNA) to examine relationships between sample traits and gene expression profiles. Our analysis focused specifically on mRNA instability index (mRNAsi) and EREG-mRNAsi [29]. Using WGCNA’s soft threshold function, we tested power values (β) from 1 to 30 to optimize network construction. We then converted the adjacency matrix into a topological overlap matrix. We identified modules through dynamic tree cutting of the gene dendrogram and merged similar modules. Using Pearson correlation analysis, we identified modules significantly associated with mRNAsi. From the resulting 14 modules, we selected two key modules (MEgreen and MEturquoise) for detailed investigation based on their module-trait correlations and statistical significance (Supplementary Table S2). We defined hub genes within these modules using strict criteria: module membership >|0.5| and gene significance >|0.3|.

### Identification and validation of BIN1 by combining proteomics and transcriptomics

We identified differentially expressed proteins by comparing tumor and normal samples from two sources: our urine proteomics dataset [26] and the proteomics dataset published by Zhang et al. [27]. Using selection criteria of *p* < 0.05 and log2FC > 0.585, we detected 2,102 differentially expressed proteins in our dataset and 416 proteins in Zhang’s dataset. By integrating these proteins with hub genes from the WGCNA MEgreen and MEturquoise modules, we identified six key genes. We then evaluated BIN1 expression differences between BLCA and normal control groups using the Wilcoxon signed-rank test.

### Association between BIN1 expression with molecular and immune characteristics

We divided BLCA patient samples into high and low BIN1 expression groups using the median expression level as the threshold. Through differential analysis with criteria of log2FC > 1.5 and *P* < 0.05, we identified 1,871 differentially expressed genes (DEGs) (Supplementary Table S3). We then performed Gene Ontology (GO), Kyoto Encyclopedia of Genes and Genomes (KEGG), and Disease Ontology (DO) enrichment analyses to understand their functional roles and pathway associations.

We analyzed the immune and molecular characteristics of the TCGA-BLCA cohort using CIBERSORT and ESTIMATE algorithms. These analyses quantified immune cell infiltration abundance, immune scores, tumor purity, ESTIMATE scores, and stromal scores. To investigate immune variations, we compared immune cell infiltration patterns and immune scores between high and low BIN1 expression groups. We also examined correlations between BIN1 expression and immune cell infiltration levels using Pearson correlation analysis.

Finally, we evaluated associations between BIN1 expression and both tumor mutation burden (TMB) score and stemness index to assess its prognostic value in BLCA.

### Cell culture

We cultured human BLCA cell lines (J82, UM-UC-3, T24, 5637, 253 J, and HT1376) in RPMI-1640 medium and human bladder epithelial cells (SV-HUC-1) in DMEM high glucose medium (Shanghai Yuanpei Biotechnology, China). Both media contained 10% fetal bovine serum (FBS, PAN Biotech, Germany). All cells were maintained at 37 °C in a humidified atmosphere with 5% CO2. The Key Laboratory of Urological Diseases, Second Hospital of Lanzhou University, provided all cell lines used in this study.

For BIN1 knockdown, we obtained lentiviral packaging vectors (psPAX2 and pMD2.G), shRNA lentiviral vector pLKO.1, and an empty lentiviral vector from the Miaoling Plasmid Sharing Platform (China). We produced shRNA lentiviruses targeting BIN1 following Addgene’s protocol. Using Sigma-Aldrich’s online tool (https://www.sigmaaldrich.cn/), we designed two shRNA sequences: sh1 (GAAACCTAAGCCAAGGTAT) and sh2 (CCTGATATCAAGTCGCATT), which were synthesized by Tsingke Biological Technology. For BIN1 overexpression, we amplified the full-length BIN1 coding sequence (CDS) from human cDNA and cloned it into the pcDNA5-flag vector using In-fusion recombination. The primer sequences used were: Forward, CGTCGAAGCCCGGGCGGATCCATGGCAGAGATGGGCAGTAAAGG; Reverse, ATCGGGCCCTCTAGACTCGAGTCATGGGACCCTCTCAGTGAAGTTCTCG. UMUC-3 and J82 cells, post-transfection, were selected using puromycin after 72 h.

### Quantitative real-time PCR analysis

We extracted total RNA from cells using TRIzol reagent and normalized RNA concentrations by spectrophotometry. Using the AJ reverse transcription kit protocol, we reverse transcribed 1 µg of total RNA into cDNA. We performed quantitative real-time PCR (qRT-PCR) using a BIO-RAD CFX-96 system and analyzed the data using the 2 − ΔΔCt method. All statistical analyses and graphs were generated using Prism 8.0 software. Primer sequences are listed in Supplementary Table S4.

### Western blot

We extracted total protein using RIPA buffer (P0013B, Beyotime, China) containing protease inhibitors and measured protein concentrations using the Bicinchoninic Acid Assay (BCA). After separating proteins by electrophoresis, we transferred them to PVDF membranes. We blocked the membranes with 6% non-fat dry milk and incubated them with primary antibodies overnight at 4 °C. Using the Odyssey^®^ imaging system and IRDye 800CW Goat anti-Rabbit IgG secondary antibody (926-32211, Li-Cor, USA), we detected and visualized the immunoblots. Details of primary antibodies are provided in Table S5.

### Growth curves

We evaluated cell growth using the CCK-8 assay following the manufacturer’s instructions. J82 and UM-UC-3 cell lines, along with their derivatives, were seeded at a density of 2 × 10³ cells/well in 96-well plates, while other cell lines were seeded at 3 × 10³ cells/well. CCK-8 reagent was added at five time points (0, 1, 2, 3, and 4 days after seeding), and the cells were incubated with the reagent for 2 h before measuring optical density (OD) at 450 nm. Relative cell proliferation was calculated as ODt/OD0, where OD0 represents the optical density at Day 0 (baseline), and ODt represents the optical density at subsequent time points (Day 1, Day 2, Day 3, or Day 4). Each experiment was performed in triplicate.

### Colony formation assay

Cells were plated at a density of 300 cells per well in 6-well plates, with the culture medium being refreshed every 3 to 4 days. Prior to collection, UMUC3, J82, and their derived cell lines were incubated for a duration of 10 days. For the purpose of colony enumeration, cells underwent fixation with 4% paraformaldehyde and were subsequently stained using a 0.1% crystal violet ammonium oxalate solution.

### Cell migration/invasion assays

We evaluated cell migration and invasion using Transwell assays. For migration assays, we seeded 20,000 cells in 200 µl serum-free 1640 medium into the upper chambers of 24-well inserts (8 μm pore size; Corning Life Sciences, USA). The lower chambers contained 1640 medium with 10% fetal bovine serum as chemoattractant. For invasion assays, we pre-coated the upper chamber membranes with Matrigel (354262, Corning, USA) for 4 h at 37 °C before cell seeding, maintaining other conditions identical to the migration assay. After 24 h, we fixed migrated or invaded cells with 4% formalin for 30 min, stained them with 0.05% crystal violet for 20 min, and quantified them using a 20× inverted microscope.

### Cell cycle detection

We harvested J82 and UMUC-3 cells in logarithmic growth phase and seeded them at 2 × 10^5 cells per well in 6-well plates. After cell attachment, we digested and resuspended the cells, followed by centrifugation at 1000 g for 5 min. The cell pellet was washed with pre-chilled PBS and fixed in 70% ethanol for 2 h. After centrifugation, we stained each sample with a mixture containing 480 µL Staining Solution, 10 µL RNase A Solution, and 10 µL PI Solution. The samples were incubated at 37 °C for 30 min in darkness and analyzed using a Beckman flow cytometer (AO-1-1102, Beckman Coulter, USA). We performed data analysis using FlowJo software (V10.8.1). All experiments were conducted in triplicate.

### TUNEL cell apoptosis detection

We seeded cells in logarithmic growth phase into 24-well plates. After 24 h of attachment, we washed cells twice with PBS and fixed them with 4% paraformaldehyde for 30 min. The cells were then washed three times with PBS, permeabilized with 0.3% Triton X-100 in PBS for 5 min at room temperature, and washed twice with PBS. We added 50 µl TUNEL assay solution (C1089, Beyotime, China) to each well and incubated for 60 min at 37 °C in darkness. After three PBS washes, we detected TUNEL-positive cells using fluorescence microscopy at 550 nm excitation and 570 nm emission wavelengths (red fluorescence).

### Tumor sphere formation assay

To assess the growth and formation of tumor spheres, UMUC-3 cells were seeded in 24-well ultralow attachment plates (Corning Inc., Corning, NY, USA) at a density of 1,000 cells per well. The medium used was serum-free DMEM/F12, enriched with 20 ng/ml human epidermal growth factor (EGF), 10 ng/ml human basic fibroblast growth factor (bFGF), 2% B27, and 1% N2 supplement (Invitrogen). Cultures were maintained in a humidified atmosphere at 37 °C and 5% CO2. After two weeks, tumor sphere formation was evaluated and quantified using an inverted microscope, with all measurements conducted in triplicate.

### Immunofluorescence

BLCA cells, numbering 5 × 10^4, were cultured on glass slides within 24-well plates for a duration of 24 h. Subsequently, the slides were transferred onto cover slides in preparation for further experimentation. The slides underwent a triple wash with PBS, followed by fixation with 4% paraformaldehyde for 10 min. Permeabilization was achieved using 0.5% Triton X-100 in PBS, after which the cells were incubated overnight at 4 °C with a primary antibody diluted 1:100 in PBS. The following day, slides were brought to room temperature and incubated with a fluorescent secondary antibody for one hour. Nuclei were stained with DAPI for five minutes, and the slides were sealed with glycerine. Immunofluorescence for the targeted proteins was observed using a positive fluorescence microscope (Olympus, Tokyo, Japan), with the entire process replicated in triplicate for accuracy.

### Proteomic analyses

UMUC-3 cells were cultured in RPMI-1640 medium containing 10% fetal bovine serum in 10 cm culture dishes. After harvesting and washing, the cells were sent to Jingjie PTM BioLab Co., Ltd. for protein profiling. For identification of differentially expressed proteins (DEPs), three biological replicates each of control and BIN1-overexpressing UMUC-3 cells were compared using four-dimensional liquid chromatography-tandem mass spectrometry (4D-LC-MS/MS) with label-free quantification (4D-LFQ). Proteins with *P* < 0.05 were defined as differentially expressed.

### Immunoprecipitation assays

Immunoprecipitation was performed using the Beaver Biological Protein A/G Immunoprecipitation Magnetic Bead Kit according to manufacturer’s instructions. BIN1-overexpressing and control UMUC-3 cells were lysed in IP buffer, sonicated in an ultrasound bath, and centrifuged at 14,000 g for 10 min. The supernatant was incubated with Protein A/G magnetic beads and specific antibodies overnight at 4 °C. After PBS washing and centrifugation, the samples were denatured in SDS loading buffer at 95 °C for 10 min, followed by western blot analysis as described above.

### SiRNA construction and cell transfection

Small interfering RNAs targeting Myc (si-Myc; sequence: GGAACUAUGACCUCGACUATT), ALDH1 (si-ALDH1A1; sequence: GGGCGACUAUUAUUAUACAAGUTT), and a negative control siRNA (si-NC; sequence: CGAACAGTCACTAGTCACGAT) were synthesized by Gemma Gene (Shanghai, China). UMUC cells were plated in six-well plates at an optimized density prior to transfection. The transfection process employed Lipo6000 reagent (Biyuntian, Shanghai, China), conducted strictly in accordance with the manufacturer’s protocol. Forty-eight hours post-transfection, the cells were harvested for protein analysis assays.

### 3D matrigel drop invasion assay

2 × 10^4^ cells per droplet were transferred into 1.5 ml EP tubes. Subsequently, the EP tubes underwent centrifugation at 1000 g for 5 min at 4°C, followed by the meticulous removal of the supernatant medium. Thawed matrigel was gently introduced to the cell pellet within the EP tube and mixed thoroughly to ensure homogeneity without the formation of any bubbles. Depositing 10 µl of the matrigel cell suspension at the center of each well in 24-well plates, a droplet was meticulously formed, free from any entrapped air. The 24-well plates were then placed in a 37 °C incubator with 5% CO_2_ for 15 min to allow the droplets to solidify. After solidification, 1 ml of complete medium was precisely applied to each well, trickling down the sidewall with precision. Subsequently, the 24-well plates were incubated and the medium was refreshed on the third day, with daily microscopic documentation capturing the developmental progression.

### Immunohistochemistry (IHC) staining and evaluation

Tissue samples were obtained from The Second Hospital of Lanzhou University. Formalin-fixed, paraffin-embedded sections were dewaxed in xylene and rehydrated through graded ethanol. Endogenous peroxidase activity was quenched by incubation in 3% hydrogen peroxide for 10 min at room temperature. Antigen retrieval was performed in Tris-EDTA buffer (pH 9.0) at 100 °C for 30 min. Sections were incubated with primary antibodies against BIN1 (1:200), ALDH1 (1:200), and NOTCH2 (1:100) overnight at 4 °C. After PBS washing, sections were incubated with secondary antibodies for 30 min at room temperature, then stained with DAB and hematoxylin. Brown coloration indicated positive staining. Protein expression was evaluated in five random fields using a combined score of staining intensity (0 = none, 1 = weak, 2 = moderate, 3 = strong, 4 = very strong) and percentage of stained cells (0 = 0%, 1 ≤ 10%, 2 ≤ 20%, 3 ≤ 30%,…, 9 ≤ 90%, 10 ≤ 100%).

### Xenograft tumor model

This study was approved by the Ethics Committee of the Second Hospital of Lanzhou University. Male athymic nude mice (4 weeks old, *n* = 10) were randomly divided into two groups of five each. After 7 days of acclimation, each mouse received bilateral subcutaneous injections of 5 × 10^6 UMUC-3 cells (Vector or BIN1) mixed with 100 µl serum-free medium and 50 µl Matrigel. Body weight and tumor size were measured weekly for 28 days. After euthanasia on day 28, tumors were collected and measured. Tumor volume was calculated as (length × width^2) × 0.5.

### Statistical analysis

Bioinformatic data analyses were performed using R software (version 4.1.0). Independent t-tests were used to compare continuous variables between groups. For categorical data, chi-square tests were performed. Additional statistical analyses were conducted using GraphPad Prism (version 9.0). Statistical significance was defined as *p* < 0.05.

## Result

### mRNAsi and EREG mRNAsi are upregulated in BLCA and correlates with prognosis

**Fig. 1:**
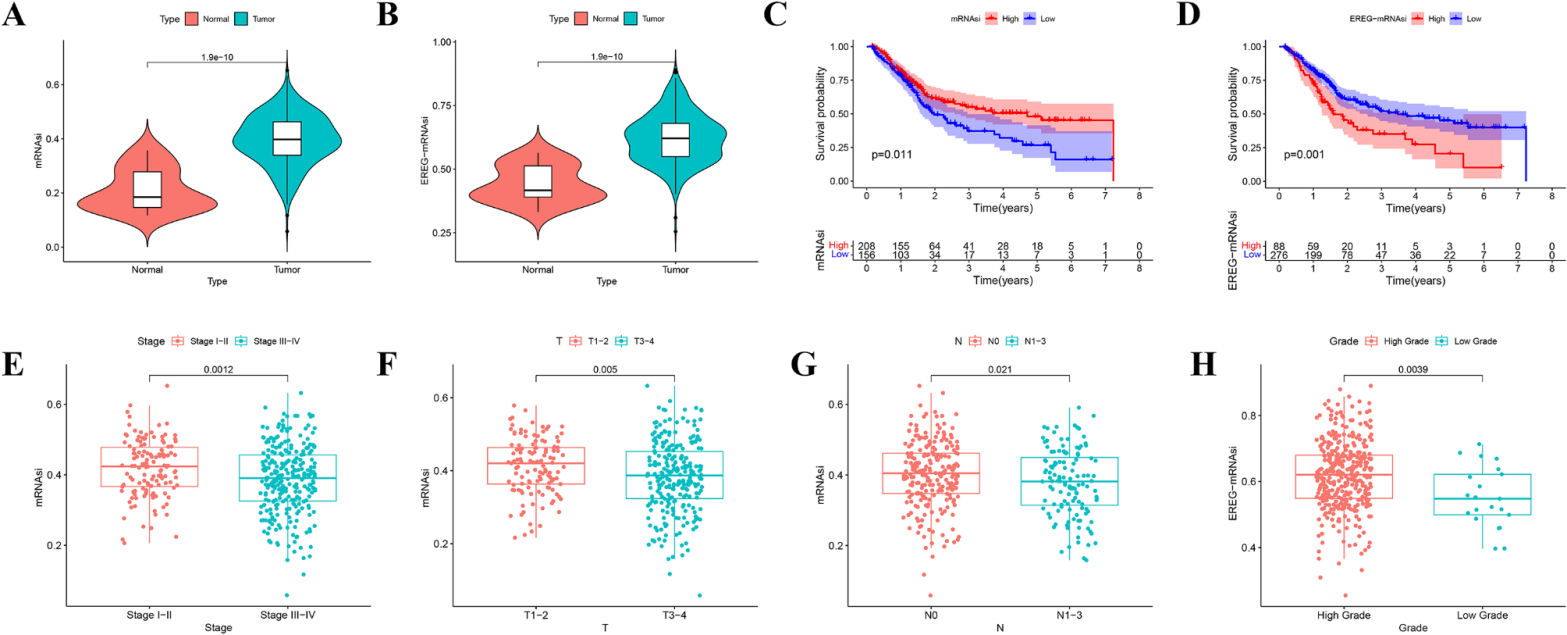
mRNAsi is closely related to the prognosis of BLCA. (**A, B**) mRNAsi and EREG-mRNAsi were upregulated in BLCA. (**C**) Kaplan-Meier overall survival curve of BLCA patients with high or low mRNAsi expression. *n* = 208 or 155, respectively. Data from the TCGA database. (**D**) Kaplan-Meier overall survival curve of BLCA patients with high or low EREG-mRNAsi expression. *n* = 88 or 275, respectively. Data from the TCGA database. Relationships between mRNAsi and pathological stage (**E**), T stage (**F**), N stage (**G**) and histological grade (**H**)

The mRNAsi and EREG-mRNAsi data for BLCA and normal samples were obtained from TCGA database [29]. Both mRNAsi and EREG-mRNAsi were higher in BLCA samples than in normal controls (Fig. 1A and B). Patients were divided into high and low mRNAsi (EREG-mRNAsi) groups using optimal cutoff values. Kaplan-Meier analysis showed better survival in patients with high mRNAsi (Fig. 1C) but worse survival in those with high EREG-mRNAsi (Fig. 1D). The mRNAsi and EREG-mRNAsi levels were also associated with clinical characteristics (Fig. 1E-H).

### Transcriptomics and proteomics data identified BIN1 is closely associated with BLCA

**Fig. 2:**
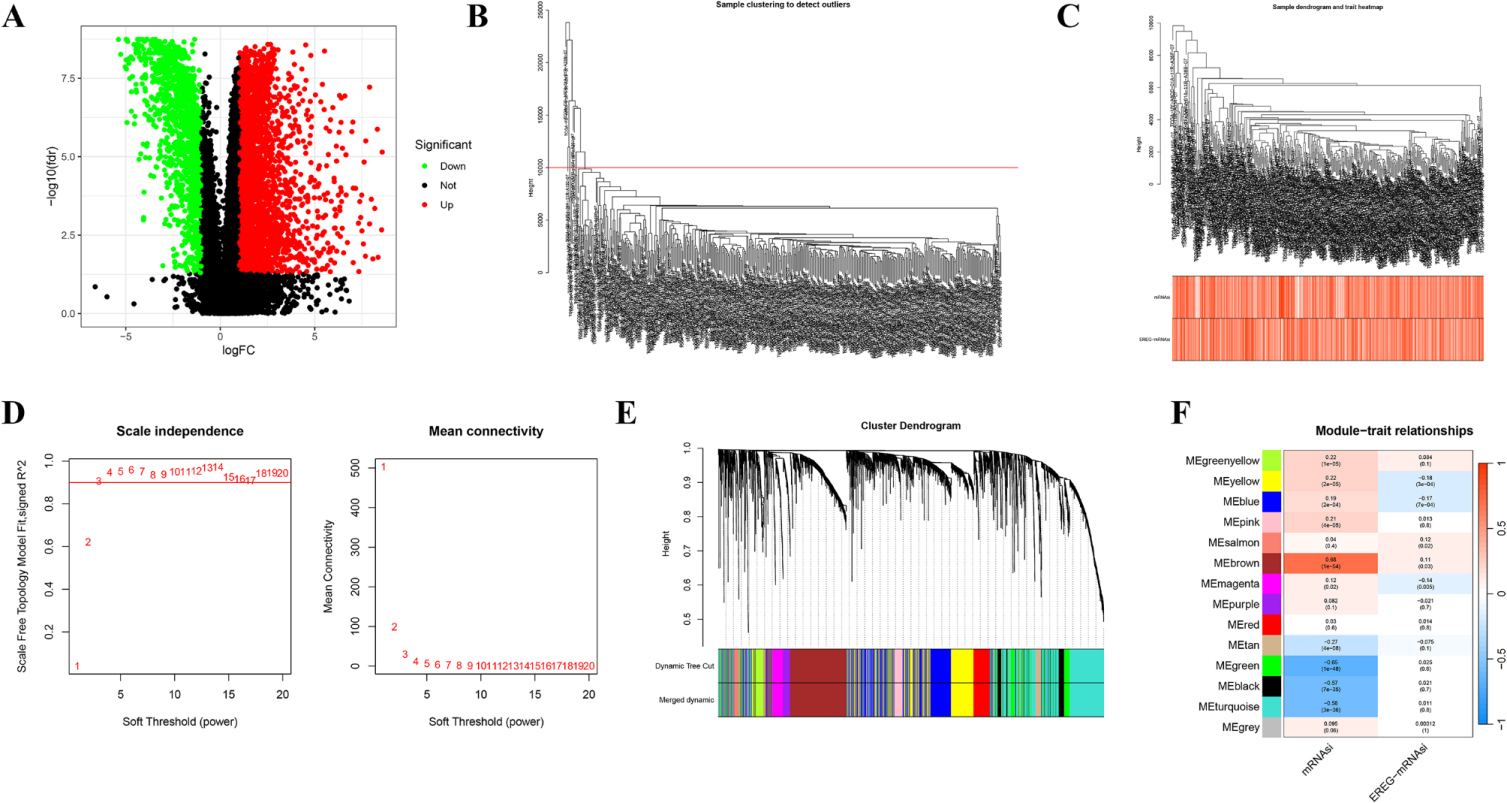
WGCNA identified BIN1 is closely associated with BLCA. (**A**) 4786 DEGs in TCGA cohort. (**B, C**) According to the sample clustering result, the sample dendrogram and trait heatmap were developmented. (**D**) Analyses of network topologies for various soft-thresholding powers through scale-free fit index and mean connectivity. (**E**) Clustering dendrogram of genes based on topological overlapping. (**F**) The heatmap showing the correlation between the gene module. The correlation coefficient in each block represented the correlation between gene module and the clinical traits

Differential gene analysis of the TCGA cohort identified 4,786 DEGs (Fig. 2A). WGCNA was performed to explore genes correlated with mRNAsi and EREG-mRNAsi. After excluding outlier samples through cluster analysis (Fig. 2B), sample dendrograms and trait heatmaps were constructed to show data structure and trait associations (Fig. 2C). A soft thresholding power of 3 was selected through WGCNA to optimize network sensitivity and specificity (Fig. 2D). The analysis identified 14 distinct gene modules within the shared expression network (Fig. 2E), and module-trait relationships were shown in Fig. 2F. Among these modules, 91 core genes in the MEgreen module and 328 in the MEturquoise module showed significant association with mRNAsi.

**Fig. 3:**
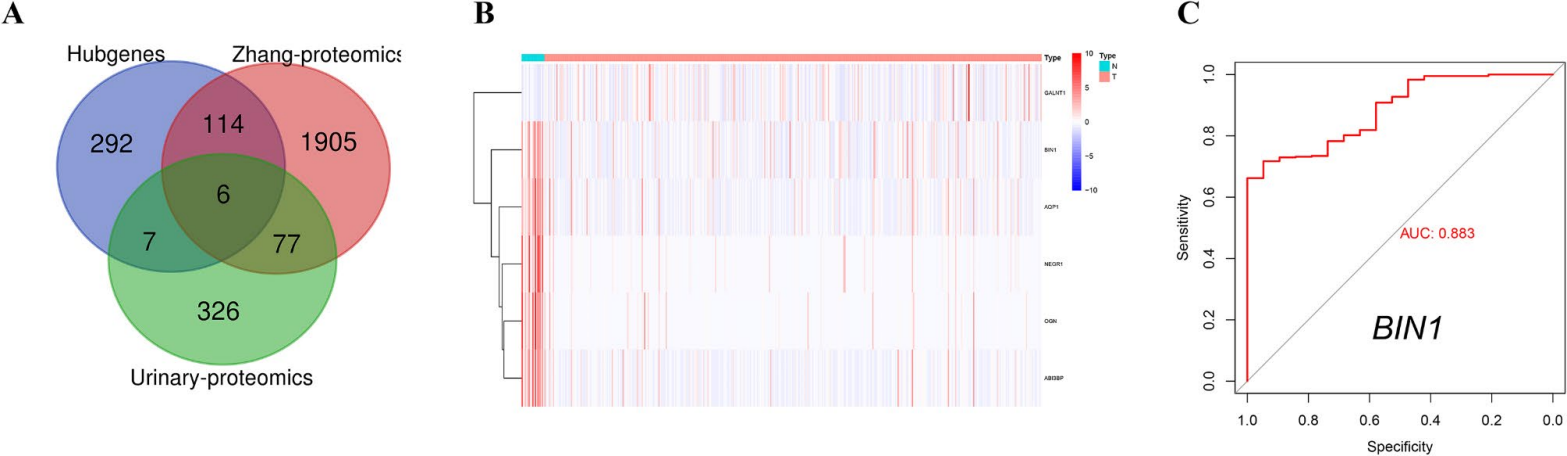
Intersecting genes of WGCNA hub genes and the proteins of Zhang et al. and Urinary proteomic datasets. (**A**) Venn Diagram showed 6 intersecting genes. (**B**) The heatmap showing the intersecting genes expression in the TCGA dataset. (**C**) ROC curve showing the predictive precision of BLCA by BIN1

Integration of hub genes from the MEgreen and MEturquoise modules (identified through WGCNA), differentially expressed proteins from Zhang et al.‘s study, and urine proteomic datasets identified six pivotal genes (Fig. 3A): BIN1, OGN, ABI3BP, AQP1, NEGR1, and GALNT1 (Fig. 3B). Based on literature review, BIN1 was selected for further investigation (Fig. 3C).

### BIN1 is downregulated in BLCA

**Fig. 4:**
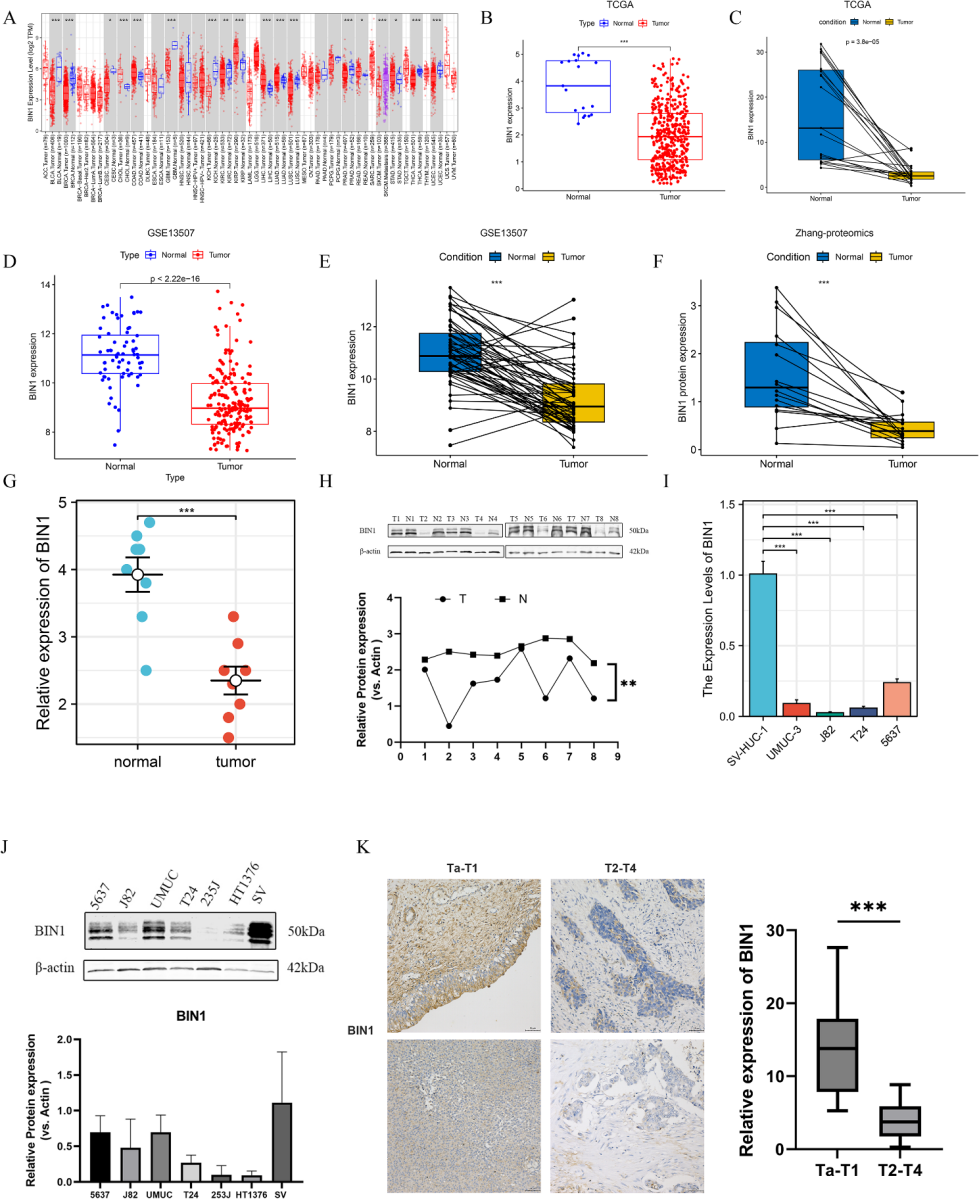
BIN1 is downregulated in BLCA tissues and cell lines. (**A**) The expression of BIN1 in pan-carcinoma as determined with the TIMER database. (**B**) The expression of BIN1 mRNA was analyzed in BLCA tissues from TCGA databases. (**C**) The pairwise analysis of BIN1 expression in tumor and normal tissue from TCGA databases. (**D**) GSE13507 dataset showed a significantly lower expression of BIN1 in BLCA tissues. The pairwise analysis of BIN1 expression in tumor and normal tissue from GSE13507 dataset (**E**) and Zhang’s database (**F**). (**G, H**) mRNA and protein levels of BIN1 in bladder tumor tissues and adjacent nontumor tissues. mRNA expression levels of target genes were normalized to those of β-actin. β-actin served as the loading control. *n* = 8 per group. (**I, J**) The expression of BIN1 in BLCA cell lines and SV-HUC-1 cells at both the transcriptional and translational levels. (**K**) BIN1 expression level is lower in higher T-stage bladder cancer. *P < 0.05, **P < 0.01, ***P < 0.001

Pan-cancer analysis through the TIMER database (http://timer.cistrome.org/) showed decreased BIN1 expression across multiple tumor types (Fig. 4A). Analysis of BLCA compared BIN1 mRNA levels between BLCA and normal samples in both TCGA and GEO cohorts. The results showed significantly reduced BIN1 mRNA expression in cancerous tissues compared with normal tissues, as shown in Fig. 4B-E. Proteomic data from Zhang et al. confirmed decreased BIN1 protein expression in BLCA tissues compared with normal tissues (Fig. 4F).

### In vitro experiments confirmed low expression of BIN1

Analysis of eight BLCA tissue samples showed significant downregulation of BIN1 in BLCA tissues compared with adjacent normal tissues at both protein (Fig. 4H) and mRNA levels (Fig. 4G). BIN1 expression was also lower in UMUC-3 and 5637 BLCA cell lines compared with SV-HUC-1 cells at both mRNA (Fig. 4I) and protein levels (Fig. 4J), consistent with the bioinformatics analyses. Although BIN1 expression was reduced in BLCA, it showed no correlation with patient overall survival or disease-free survival. IHC analysis of tumor sections from 50 BLCA patients revealed decreased BIN1 expression in higher T-stage BLCA (Fig. 4K).

### Functional analysis

**Fig. 5:**
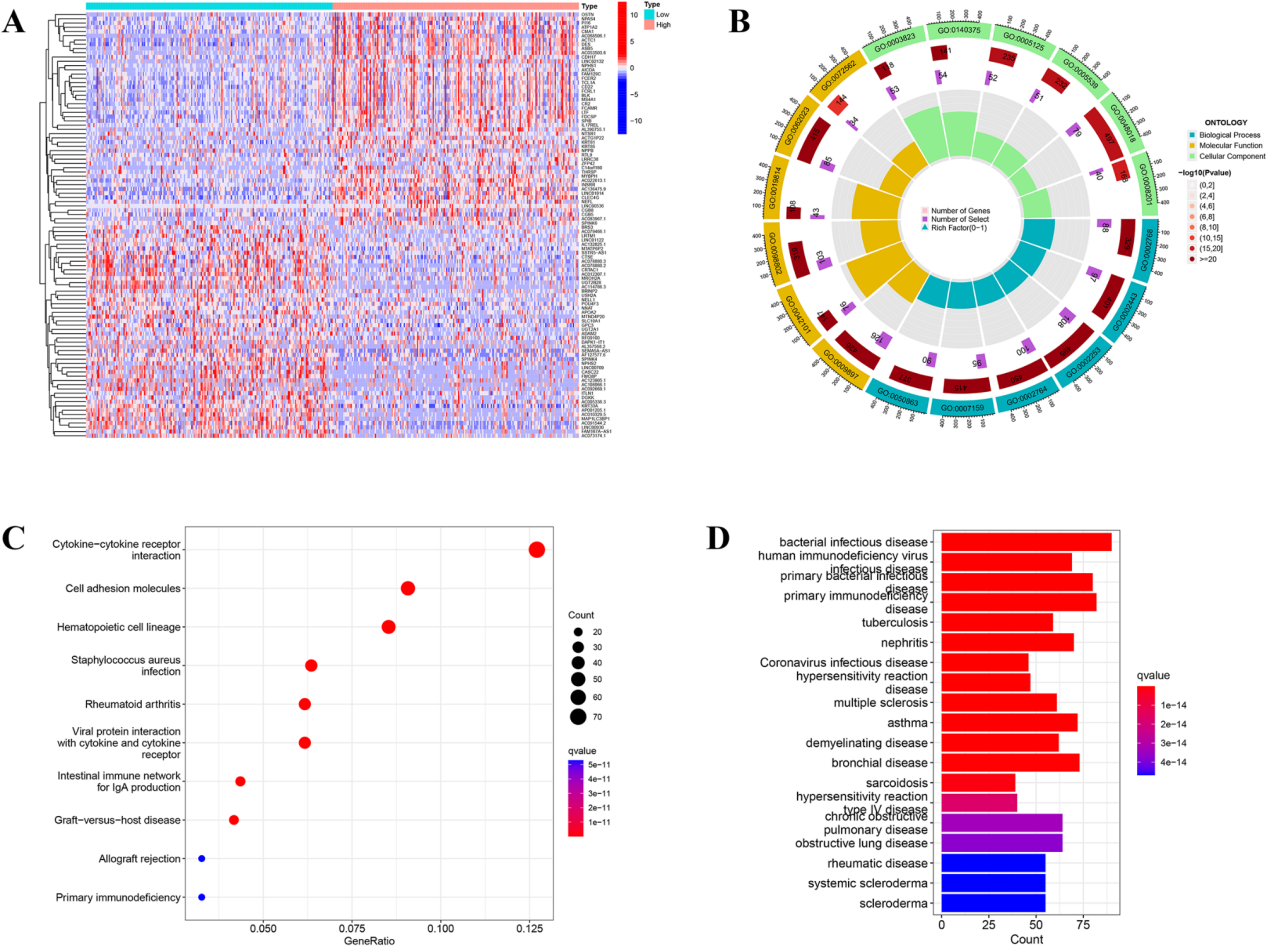
Functional analysis of the BIN1-defined subgroups. (**A**) The heatmap showing the differentially expressed genes expression on the basis of the median BIN1 expression in the TCGA dataset. (**B**) GO enrichment analysis showed the enriched biological processes(BP), cell components(CC) and molecular functions(MF) associated with the DEGs on the basis of median BIN1 expression. (**C**) KEGG pathway analysis showed the enriched signaling pathways associated with the DEGs on the basis of median BIN1 expression. (**D**) DO enrichment analysis

To delineate the genetic landscape associated with BIN1 expression in BLCA, patients were stratified into high and low BIN1 expression groups, with the median expression value serving as the cutoff. This stratification identified 1,871 genes with statistically significant differential expression (Supplementary Table S3), with the expression patterns of the top 50 DEGs shown in Fig. 5A. To investigate the functional implications of these DEGs in BLCA, GO and KEGG pathway enrichment analyses were performed. GO enrichment analysis revealed multiple significantly enriched gene sets (detailed in Supplementary Table S6), as illustrated in Fig. 5B. The three primary pathways identified through KEGG pathway analysis included Cytokine-Cytokine receptor interaction, Cell adhesion molecules, and Hematopoietic cell lineage (Fig. 5C). Disease Ontology (DO) enrichment analysis indicated that the DEGs predominantly contributed to conditions such as bacterial infectious diseases, human immunodeficiency virus infectious diseases, and primary bacterial infectious diseases (Fig. 5D), offering insights into the molecular underpinnings and potential therapeutic targets within BLCA.

### Immune and molecule characteristics of BIN1 high and low-expression groups

**Fig. 6:**
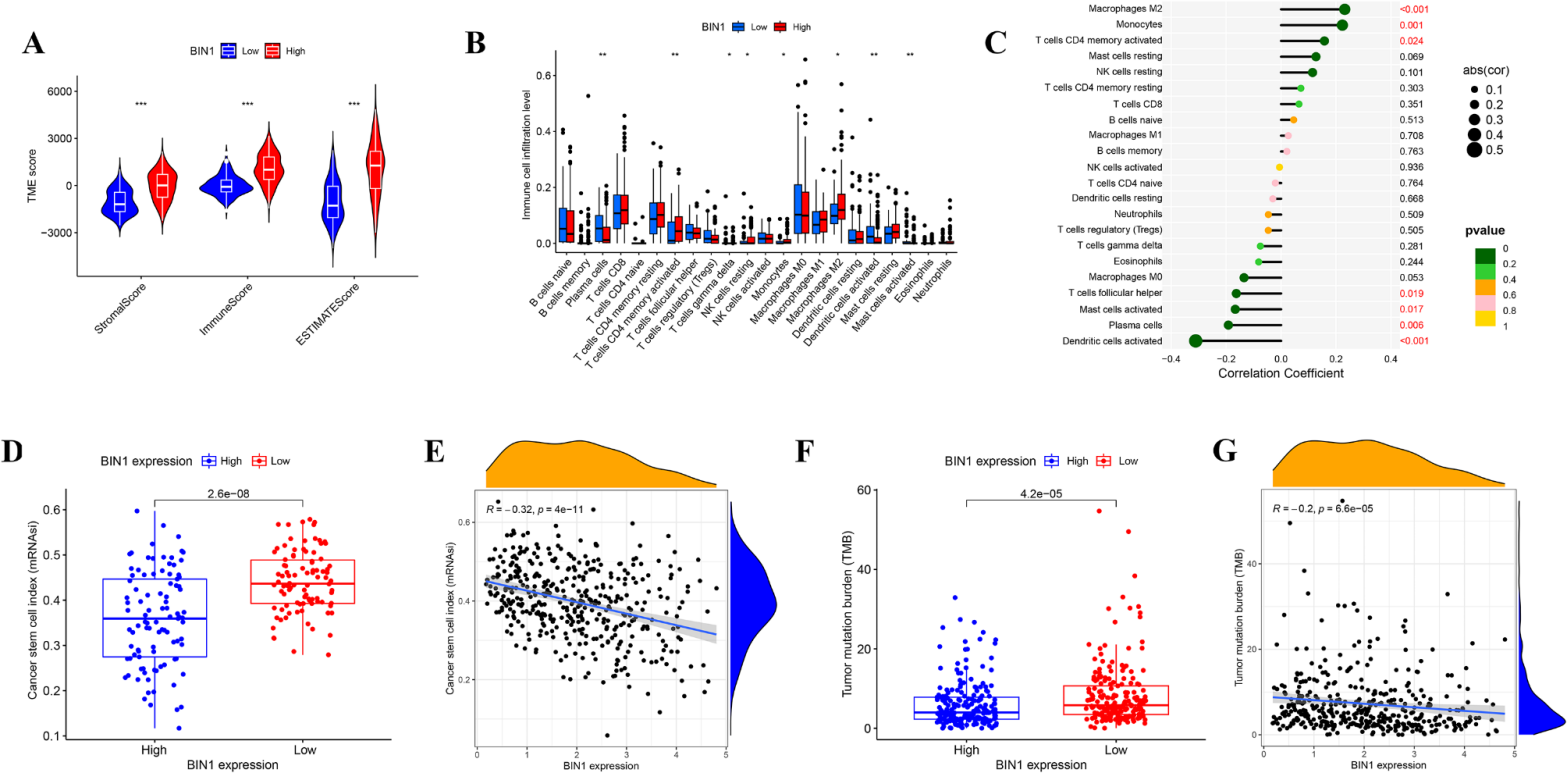
Immune and molecule characteristics of BIN1-defined subgroups (**A**) There was a statistical difference in Stromal score, Immune score and ESTIMATE score between the *BIN1* low-expression and high-expression group. (**B**) The difference of immune cells infiltration levels between the BIN1-defined subgroups. (**C**) The correlation between *BIN1* expression and the immune infiltrating cells. The BIN1 low-expression group had the higher stem cell index than that of the BIN1 high-expression group (**D**) and BIN1 expression was negatively correlated with the stem cell index (**E**). The BIN1 low-expression group had the higher TMB than that of the BIN1 high-expression group (**F**) and BIN1 expression was negatively correlated with the TMB (**G**). * represents *P* value < 0.05, ** represents *P* value < 0.01, and *** represents *P* value < 0.001, **** represents *P* value < 0.0001

BLCA patients were stratified into high and low BIN1 expression groups based on median expression level as the cutoff. The BIN1 high expression group showed significantly elevated stromal score, immune score, and ESTIMATE score (Fig. 6A). Immune cell profiling revealed enrichment of plasma cells, activated dendritic cells, and activated mast cells in the BIN1 low expression group, while CD4 memory activated T cells, resting NK cells, monocytes, and M2 macrophages were increased in the high BIN1 expression group (Fig. 6B). Correlation analyses confirmed these findings and showed consistency with the differential analysis results (Fig. 6C). The CSCs index was higher in the low BIN1 expression group, showing an inverse correlation between BIN1 expression and CSCs index (Fig. 6D and **E**). TMB was higher in the low BIN1 expression group compared with the high expression group (Fig. 6F), and showed negative correlation with BIN1 expression (Fig. 6G).

### Overexpression of BIN1 reduced proliferation, invasion, migration as well as epithelial-mesenchymal transition (EMT) and induced apoptosis in BLCA cells

**Fig. 7:**
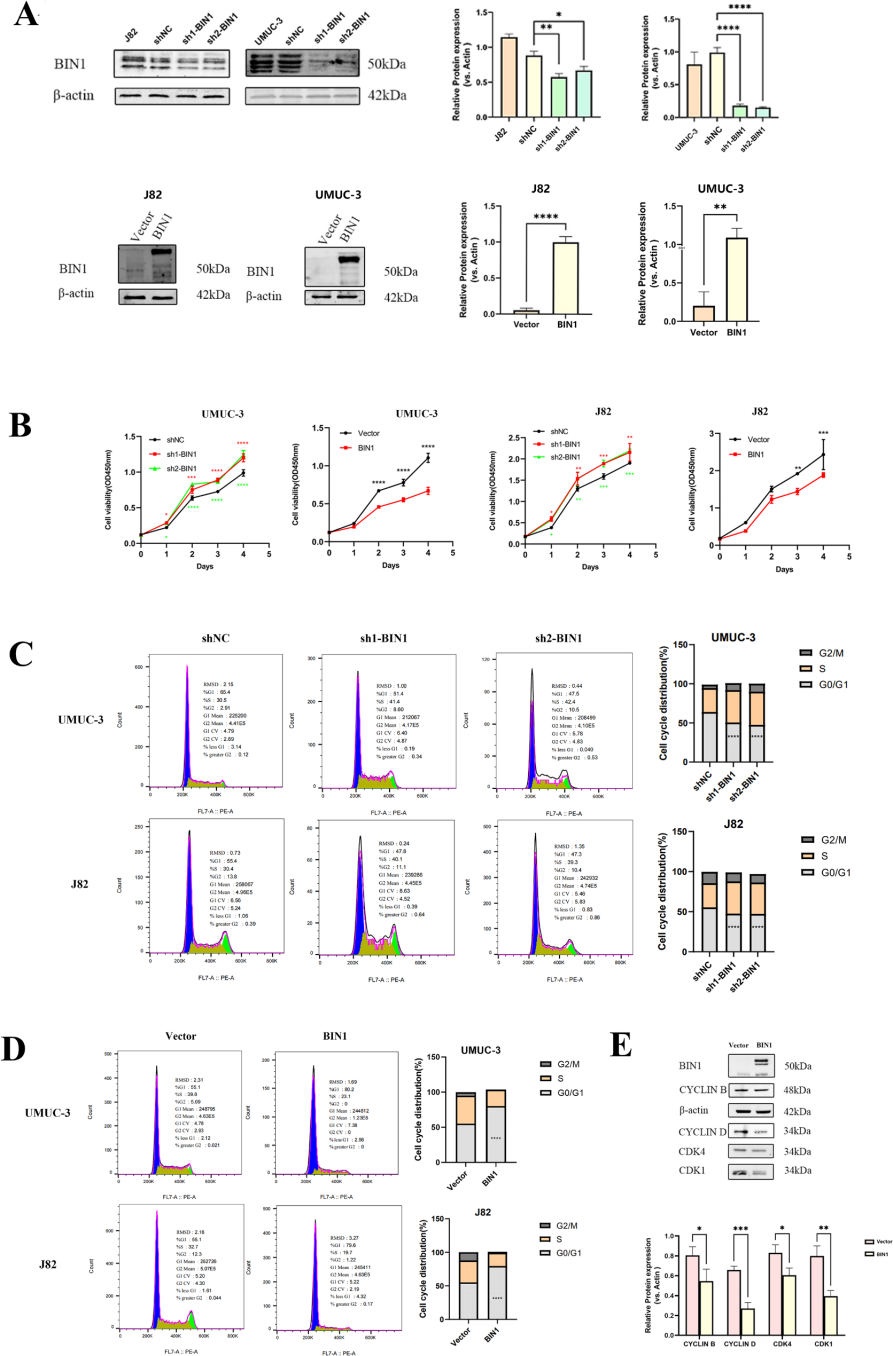
Expression of BIN1 is related to the proliferative ability of BLCA cells. (**A**) The efficiency of knocking down and overexpressing BIN1 was examined by Western blot in UMUC-3 and J82 cells. (**B**) Results of the CCK8 assay of the UMUC/J82-BIN1-OE, UMUC/J82-BIN1-sh and their control cell lines. (**C, D**) Flow cytometry analysis revealed that cell cycle arrest at G0/G1 phase when BIN1 overexpression.(**E**) The expression of cell cycle-related proteins CyclinB, CyclinD, CDK4, and CDK1 also decreased after BIN1 overexpression. * represents *P* value < 0.05, ** represents *P* value < 0.01, and *** represents *P* value < 0.001, **** represents *P* value < 0.0001

BIN1 mRNA levels were manipulated through knockdown and overexpression in UMUC-3 and J82 cells to investigate its functional role in BLCA. Western blot analysis confirmed the efficacy of these manipulations (Fig. 7A). CCK8 cell viability assays showed that BIN1 expression negatively affected BLCA cell proliferation over time (Fig. 7B). Flow cytometry analysis showed cell cycle arrest at the G0/G1 phase after BIN1 overexpression (Fig. 7C and D). Analysis of cell cycle regulatory proteins revealed decreased levels of Cyclin B, Cyclin D, CDK4, and CDK1 following BIN1 overexpression (Fig. 7E). These results indicate that BIN1 suppresses BLCA cell proliferation.

**Fig. 8:**
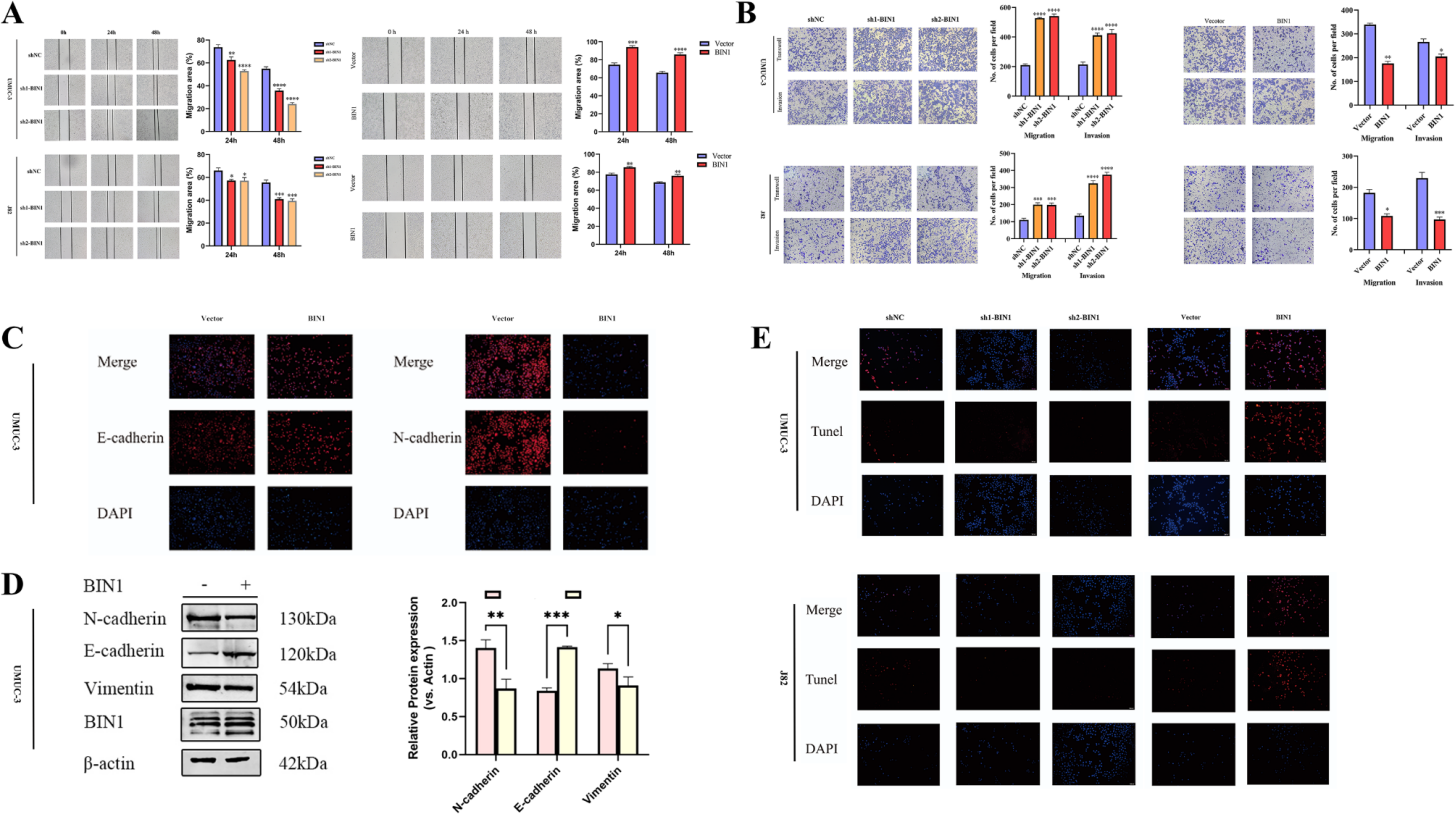
BIN1 affects migration, invasion, EMT and apoptosis of BLCA cell lines. UMUC and J82 cells were used to determine cell invasion and migration by wound-healing (**A**) and transwell assays (**B**). (**C**) The results of immunofluorescence assays for E-cadherin and N-cadherin expression were also determined in UMUC-3 cells and BIN1 overexpresssed UMUC-3 cells. Scale bars, 100 μm.(**D**) The expression of mesenchymal markers (N-cadherin, vimentin) also decreased after BIN1 overexpression. (**E**) Overexpression of BIN1 had increased levels of apoptosis, while shBIN1 decreased in TUNEL assays. * represents *P* value < 0.05, ** represents *P* value < 0.01, and *** represents *P* value < 0.001, **** represents *P* value < 0.0001

**Fig. 9:**
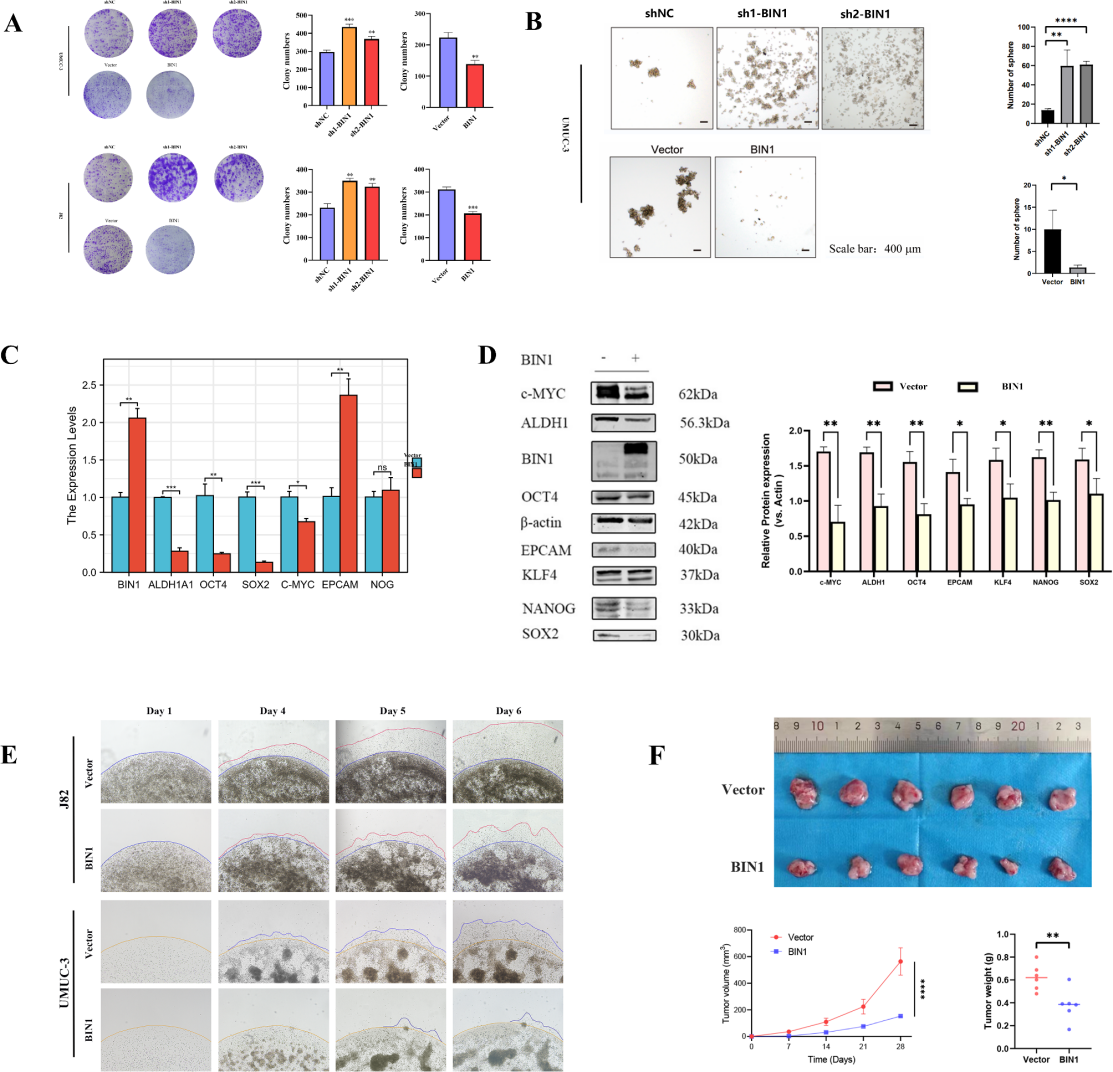
BIN1 inhibits the stemness of BLCA cells. (**A**) Images of colony formation and its statistical results of UMUC and J82 cells in the indicated groups. (**B**) Images of tumor sphere formation of UMUC cells in the indicated groups. Scale bars, 400 μm. (**C**) mRNA levels of CSC related molecules (Myc, ALDH1, OCT4, EPCAM, KLF4, NANOG, SOX2) in the indicated groups. mRNA expression levels of target genes were normalized to those of β-actin. (**D**) Protein levels of CSC related molecules (Myc, ALDH1, OCT4, EPCAM, KLF4, NANOG, SOX2) in the indicated groups. β-actin served as the loading control. Results are expressed as mean ± SD. (**E**) The 3D Matrigel drop invasion assay indicates that the invasion capability of BLCA cells diminishes when BIN1 is overexpressed. (**F**) Tumors were obtained from nude mice in the BIN1 group and vector groups, the tumor volume changes in the nude mice were measured every 7 days. Meanwhile, the wet weights of tumors in the BIN1 group and vector groups were measured and compared after the mice were sacrificed at the end of the experiment. * represents P value < 0.05, ** represents P value < 0.01, and *** represents P value < 0.001

BIN1 overexpression inhibited BLCA cell migration and invasion in vitro, as demonstrated by transwell assays and wound-healing assays in J82 and UMUC-3 cell lines. BIN1 knockdown increased wound healing rate, indicating enhanced cell migration, while its overexpression decreased cell migration (Fig. 8A). Migration assays showed similar results, and invasion assays demonstrated reduced invasive capacity of BLCA cells following BIN1 overexpression (Figs. 8B and 9E).

Furthermore, the suppression of EMT through BIN1 overexpression marks a novel discovery in BLCA research. The regulatory impact of BIN1 on EMT was evidenced by the upregulation of epithelial markers such as E-cadherin and the concomitant downregulation of mesenchymal markers including N-cadherin and vimentin in UMUC-3 and J82 cells (Fig. 8C and D). This regulatory mechanism demonstrated BIN1’s capacity to suppress the phenotypic conversion critical for metastasis.

TUNEL-based apoptosis assays revealed a significant increase in spontaneous apoptosis in BIN1-overexpressing UMUC-3 and J82 cells at 24 h, compared with decreased apoptosis levels in cells with silenced BIN1 (Fig. 8E). These findings demonstrated that restored BIN1 expression in BLCA cells significantly inhibited proliferation while promoting apoptosis, providing evidence of BIN1’s tumor-suppressive function in BLCA pathophysiology.

### Overexpression of BIN1 inhibits the stemness of BLCA cells

The significance of CSCs in tumorigenesis, recurrence, and metastasis, along with their self-renewal capacity and tumorigenic potential, has gained recognition in oncological research. CSCs contribute to malignant progression and therapy resistance in cancer. This study investigated the effects of BIN1 on CSCs properties in BLCA models. Colony formation and tumor sphere formation assays showed inhibited colony (Fig. 9A) and sphere-forming (Fig. 9B) capacities after BIN1 overexpression in UMUC-3 cells. Key CSCs markers, including ALDH1, Myc, NANOG, OCT4, SOX2, EPCAM, and KLF4, were downregulated in BIN1-overexpressing cells compared to control groups (Fig. 9C and D). These results indicate that BIN1 overexpression reduces the CSCs phenotype in BLCA cells.

### BIN1 overexpress attenuated the tumorigenicity of BLCA in vivo

BIN1’s tumorigenic potential was evaluated in vivo using a xenograft model. UMUC-3 cells with BIN1 overexpression or control cells were injected into athymic nude mice. Tumor volume and body weight were measured weekly for 28 days, followed by mouse euthanization and tumor collection. Figure 9F shows representative tumor images. BIN1-overexpressing tumors grew more slowly than control tumors, and showed lower tumor weights.

### BIN1-Mediated ALDH1/NOTCH signaling pathway

**Fig. 10:**
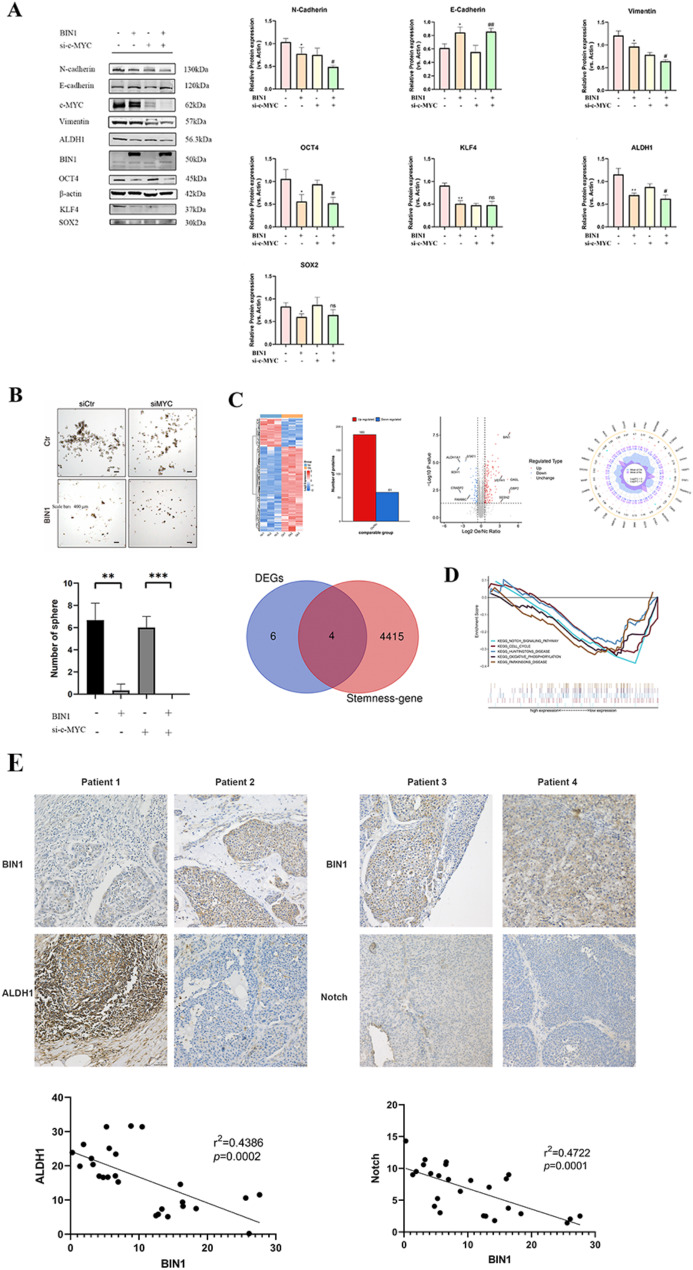
BIN1 regulates the NOTCH signaling pathway though ALDH1. (**A**) Western blot was conducted to evaluate the expression of CSC related molecules (Myc, ALDH1, OCT4, KLF4, SOX2) and EMT protein (E-cadherin, N-cadherin, Vimentin) in UMUC-3 and overexpressed BIN1 UMUC-3 cells after treatment with si-Myc. (**B**) Sphere-formation in BLCA cells showed consistent differences. Scale bars, 400 μm.(**C**) Intersecting genes of differential expressed proteins (the BIN1 group and the vector group) and stemness-genes. (**D**) GSEA analysis was performed on the sequencing data to explore the pathways related to BIN1 low expression. (**E**) The expression of ALDH1 shows a negative linear correlation with BIN1 level, and a similar trend was also observed in the expression of NOTCH. * represents *P* value < 0.05, ** represents *P* value < 0.01, and *** represents *P* value < 0.001, **** represents *P* value < 0.0001

BIN1, an interacting partner for the nuclear transcription factor Myc, influences BLCA cellular dynamics. To determine whether BIN1’s effect on BLCA cell sphere-formation occurs through Myc, we silenced Myc expression in control and BIN1-overexpressing UMUC-3 cells using siRNA. Myc suppression reduced tumor sphere formation in both cell types. However, control UMUC-3 cells formed more spheres than BIN1-overexpressing cells even after Myc knockdown. Analysis showed different expression levels of stemness markers including ALDH1A1, SOX2, OCT4, and KLF4 (Fig. 10A and B). These results indicate that BIN1 suppresses malignant BLCA cell proliferation through both Myc-dependent and Myc-independent pathways.

To understand BIN1’s regulation of the CSCs phenotype in BLCA cells, we performed proteomic differential analysis between control and BIN1-overexpressing UMUC-3 cells. The analysis identified 244 differentially expressed proteins, including the CSCs marker ALDH1 (Fig. 10C). Enrichment analysis of these proteins indicated that BIN1 affects the NOTCH signaling pathway (Fig. 10D), which regulates stem cell maintenance, stemness, differentiation, and terminal differentiation induction across tissues. Because ALDH1 promotes tumor progression through NOTCH signaling, we investigated the interactions between BIN1 and these molecules in BLCA. Analysis of BLCA patient tissues showed that ALDH1 expression negatively correlates with BIN1 levels, with NOTCH expression showing a similar pattern (Fig. 10E).

**Fig. 11:**
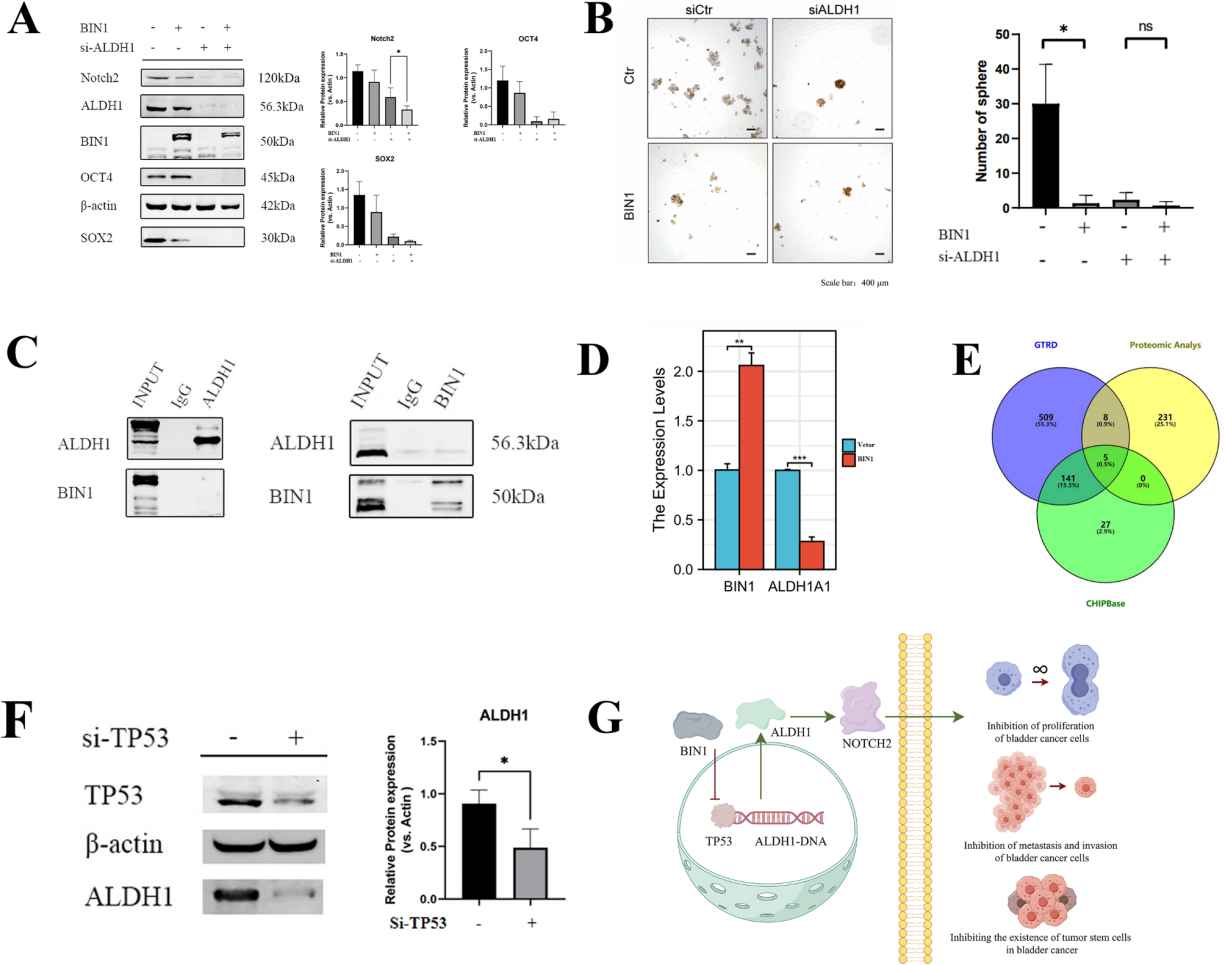
BIN1 impacts transcription of ALDH1 via TP53. (**A**) CSC related molecules (Myc, ALDH1, OCT4, KLF4, SOX2) and EMT protein (E-cadherin, N-cadherin, Vimentin) in UMUC-3 and overexpressed BIN1 UMUC-3 cells after treatment with si-ALDH1. (**B**) ALDH1 knockdown eliminated the stemness of UMUC-3 cells and UMUC-3 cells overexpressing BIN1.Almost no sphere-forming cells in either group. (**C**) Immunoprecipitation assays shows BIN1 did not alter ALDH1 protein stability. (**D**) ALDH1 transcriptional activity was inhibited after overexpression of BIN1. (**E**) Five transcription factors are predicted as the potential upstream regulators of ALDH1 through GTRD, CHIPBase, and the proteomics that we measured. (**F**) si-TP53 decreased ALDH1 expression in UMUC-3 cells. (**G**) Schematic diagram of the mechanism by which BIN1 regulates bladder cancer dryness through the ALDH1/NOTCH pathway. * represents *P* value < 0.05, ** represents *P* value < 0.01, and *** represents *P* value < 0.001, **** represents *P* value < 0.0001

Experiments validated our hypothesis that BIN1 affects BLCA cell stemness through the ALDH1/NOTCH axis. ALDH1 depletion reduced stemness in both control and BIN1-overexpressing UMUC-3 cells, preventing sphere formation (Fig. 11A and B). SOX2 and OCT4 levels decreased, and NOTCH pathway activity was inhibited (Fig. 11A). These results show that BIN1 regulates BLCA cell stemness through the ALDH1/NOTCH signaling pathway.

### BIN1 impacts transcription of ALDH1 via TP53

In investigating the interactions between BIN1 and ALDH1 in BLCA stemness, our studies revealed several findings. In UMUC-3 cells, BIN1 overexpression decreased both mRNA and protein levels of ALDH1. Our experiments showed that while silencing either ALDH1 or Myc reduced the sphere-forming capacity of UMUC-3 cells, only ALDH1 knockdown completely prevented tumor sphere formation. These observations demonstrated ALDH1’s central role in mediating the stemness properties of BLCA cells through BIN1 modulation. We then examined how the BIN1-ALDH1 axis influenced BLCA stem cell characteristics. While BIN1 suppressed ALDH1 transcriptional activity, it did not affect ALDH1 protein stability (Fig. 11C), suggesting transcriptional regulation (Fig. 11D). To identify key transcription factors (TFs) controlling ALDH1 expression in BLCA, we silenced five TFs known to promote ALDH1 (Fig. 11E). Among these, only TP53 knockdown decreased ALDH1 expression. Analysis showed that silencing TP53 also reduced ALDH1 transcriptional activity in cells overexpressing BIN1 (Fig. 11F). These findings demonstrated that TP53 and ALDH1 work together in the BIN1-mediated stemness regulation of BLCA cells.

## Discussion

BLCA is the most prevalent malignancy of the urinary system and is among the most economically burdensome cancers worldwide because of its lack of disease-specific features and frequent recurrences [30, 31]. While cystoscopic biopsy is the most reliable method for BLCA diagnosis, repeated cystoscopy and treatment create significant economic burden. However, studies on noninvasive early diagnostic evaluations and specific markers for BLCA remain limited [32]. The US Food and Drug Administration (FDA) has approved six urine tests for clinical use with cystoscopy: NMP22 ELISA, NMP22 BladderChek, UroVysion, immune cells (UCyt+), BTA-TRAK, and BTA-STAT. None of these tests has gained broad acceptance for BLCA diagnosis or follow-up [33]. Urine markers have been a critical area of BLCA research over recent decades [4]. Because urine flows through the bladder, urinary markers may provide evidence for early BLCA detection and recurrence monitoring. Our group conducted the first urinary proteomics tests for early-stage BLCA, performing quantitative analysis of urinary protein expression profiles in BLCA patients and healthy controls using Label-free/DIA [26]. The analysis identified 725 differential proteins: 350 with increased expression and 375 with decreased expression in BLCA. We cross-referenced these proteins with another database using bioinformatics methods. Our target protein, BIN1, showed significantly lower expression in BLCA patient urine compared to healthy controls. The mechanism by which BIN1 affects BLCA development and progression needs further investigation. Future experimental studies addressing these questions will strengthen the theoretical foundation for early screening, recurrence monitoring, and treatment of BLCA.

Patients with metastatic BLCA often have poor prognosis, mainly due to the lack of effective treatments. Distant metastases, particularly to the lungs, and chemoresistance are the primary causes of mortality in BLCA [34]. Therefore, understanding the molecular mechanisms of BLCA progression is crucial. BIN1, first identified as a pro-apoptotic tumor suppressor that interacts with and inhibits the oncogenic transcription factor MYC, has complex physiological roles. These include endocytosis, membrane cycling, cytoskeleton regulation, DNA repair, cell cycle control, and apoptosis. BIN1 expression is reduced or absent in many human cancers, including esophageal, gastric, neuroblastoma, breast, lung, colorectal, prostate, pancreatic cancers, and malignant pleural mesothelioma [18, 35–39]., making it an important focus in cancer research. The mechanism by which BIN1 suppresses tumors in various cancers remains unclear and has drawn our attention. In breast cancer, reduced BIN1 expression combined with RAS activation accelerates tumor growth, establishing BIN1 as a key inhibitor of breast cancer development and progression [37]. In cutaneous T-cell lymphoma, researchers have observed decreased BIN1 expression alongside increased c-FLIP expression. BIN1 loss may contribute to CTCL pathogenesis by promoting apoptosis resistance [40]. In pancreatic cancer, BIN1 acts as a macrophage phenotype switching associated gene and serves as an independent risk indicator for prognosis [38]. Our research shows, for the first time, that BIN1 is underexpressed in BLCA and functions as a tumor suppressor gene. This study demonstrates BIN1’s tumor-suppressive role in BLCA, advancing our understanding of this malignancy.

BIN1 suppresses cancer cell proliferation by promoting apoptosis in BLCA cells. Previous studies have shown that BIN1 overexpression enhances apoptosis in malignant cells [13, 40]. and inhibits malignant transformation through both caspase-dependent and caspase-independent apoptotic pathways [15, 16, 41]. A BIN1 inhibits tumor cell proliferation through both Myc-dependent and Myc-independent mechanisms [14, 15]. E K Cassimere et al. [42] found that E2F1 transcriptionally activates BIN1, which then mediates E2F1-induced apoptosis in response to DNA damage through Myc interaction. E2F1 depletion reduces BIN1 expression, demonstrating BIN1’s role as a Myc inhibitor in Rb/E2F1-dependent G1 phase arrest. In our study of the J82 cell line, flow cytometric apoptosis assays and TUNEL assays showed that BIN1 overexpression increased apoptosis in BLCA cells and induced G1 phase cell cycle arrest. In contrast, shBIN1 cells showed reduced apoptosis and increased S phase cell population. The mechanisms by which BIN1 regulates apoptosis in BLCA, and whether this regulation depends on Myc or E2F1, require further investigation.

On the other hand, CSCs are subsets of highly tumorigenic cancer cells with self-renewal capability and have been identified in various cancer types [43]. CSCs are pivotal in tumor initiation and maintenance, contributing to distant metastasis and poor prognosis. The EMT process, observed within CSCs populations, is driven by CSCs [44], making CSCs-targeted therapies a promising approach for treating metastatic cancers. Our findings indicate a negative correlation between BIN1 expression and the CSC index, suggesting BIN1’s critical role in modulating tumor cell stemness. BIN1 overexpression correlated with reduced expression of key stemness and EMT markers, including Myc, ALDH1, OCT4, EPCAM, KLF4, NANOG, SOX2, and decreased tumor sphere-formation capabilities, suggesting EMT inhibition. Because BIN1 was initially identified as a binding partner for the nuclear transcription factor Myc [14] - a notable CSCs marker - we hypothesized that BIN1 might influence BLCA cell stemness through Myc modulation. However, experimental evidence did not support this hypothesis. When comparing UMUC-3 cells with and without BIN1 overexpression, Myc depletion reduced tumor sphere-formation significantly. Under siMyc conditions, sphere-formation levels in control cells remained higher than in cells with BIN1 overexpression, indicating that Myc does not primarily mediate BIN1’s stemness regulation in BLCA cells. This raises the question: What are the main factors involved in BIN1-regulated stemness in BLCA cells?

Proteomic analysis of UMUC-3 cells with and without BIN1 overexpression identified 244 differential proteins, with 61 showing negative correlation with BIN1 expression. Functional enrichment analyses and literature review identified ALDH1 as significantly associated with BIN1 expression. ALDH1 serves as a key marker of CSCs in multiple cancers [45, 46]. Experimental validation confirmed that BIN1 suppresses cancer cell proliferation, migration, invasion, and stem cell characteristics by targeting ALDH1. BIN1 reduced ALDH1 transcriptional activity in UMUC-3 cells without affecting protein stability. To understand the mechanism, we identified five potential ALDH1 transcription factors by comparing our proteomic data with existing databases. Only TP53 silencing decreased ALDH1 expression, suggesting that BIN1 represses ALDH1 transcription through TP53. This finding provides new insights into molecular interactions affecting cancer cell stemness and suggests a potential therapeutic target.

The NOTCH signaling pathway regulates cell fate determination, survival, and proliferation, with dual roles in carcinogenesis and tumor suppression based on tissue type and cellular context [47]. The role of NOTCH signaling in BLCA remains unclear, though evidence shows distinct functions of its receptors. NOTCH1 activation inhibits proliferation in BLCA cell lines, suggesting its role as a tumor suppressor [48]. In contrast, NOTCH2 acts as an oncogene, promoting cell proliferation and metastasis through EMT, cell cycle progression, and stem cell maintenance [49]. This contrast between NOTCH1 and NOTCH2 in BLCA progression highlights the NOTCH pathway’s potential as a therapeutic target, especially in CSCs research [49]. Our study shows that BIN1 overexpression suppresses BLCA stemness by regulating the ALDH1/NOTCH2 axis. Proteomic analysis and Gene Set Enrichment Analysis (GSEA) showed downregulation of the “KEGG NOTCH SIGNALING PATHWAY” with increased BIN1 expression. As a tumor suppressor, BIN1 affects tumor proliferation, metastasis, and stemness through the ALDH1/NOTCH2 pathway. ALDH1 silencing reduced NOTCH2, SOX2, and OCT4 expression and decreased sphere-formation in both UMUC-3 cells and cells overexpressing BIN1, indicating that BIN1 regulates ALDH1A1/NOTCH signaling through TP53. The mechanism of BIN1’s influence on TP53 and the relationship between ALDH1 and NOTCH2 require further study.

## Conclusion

This study confirms BIN1 acts as a tumor suppressor gene, inhibiting BLCA proliferation, metastasis, and differentiation through the ALDH1/NOTCH signaling pathway. ALDH1, a critical stem cell marker in BLCA, regulates this malignancy through the NOTCH2 signaling pathway (Fig. 11G). Thus, BIN1 may serve as a novel prognostic marker and therapeutic target for BLCA, particularly in its metastatic form.

## Electronic supplementary material

Below is the link to the electronic supplementary material.

Supplementary Material 1

Supplementary Material 2

Supplementary Material 3

Supplementary Material 4

Supplementary Material 5

Supplementary Material 6

## Data Availability

No datasets were generated or analysed during the current study.

## References

[1] Richters A, Aben KKH, Kiemeney L. The global burden of urinary bladder cancer: an update. World J Urol. 2020;38(8):1895–904. 31676912 10.1007/s00345-019-02984-4 PMC7363726

[2] Dancik GM, Owens CR, Iczkowski KA, Theodorescu D. A cell of origin gene signature indicates human bladder cancer has distinct cellular progenitors. Stem Cells. 2014;32(4):974–82. 24357085 10.1002/stem.1625 PMC3960367

[3] Matuszczak M, Salagierski M. Diagnostic and prognostic potential of biomarkers CYFRA 21.1, ERCC1, p53, FGFR3 and TATI in bladder cancers. Int J Mol Sci 2020, 21(9). 10.3390/ijms21093360 PMC7247579 32397531

[4] Mertens LS, Claps F, Mayr R, Bostrom PJ, Shariat SF, Zwarthoff EC, et al. Prognostic markers in invasive bladder cancer: FGFR3 mutation status versus P53 and KI-67 expression: a multi-center, multi-laboratory analysis in 1058 radical cystectomy patients. Urol Oncol. 2022;40(3):110. e111-110 e119. 10.1016/j.urolonc.2021.10.010 34906411

[5] Witjes JA, Bruins HM, Cathomas R, Comperat EM, Cowan NC, Gakis G, et al. European Association of Urology Guidelines on muscle-invasive and metastatic bladder Cancer: Summary of the 2020 guidelines. Eur Urol. 2021;79(1):82–104. 32360052 10.1016/j.eururo.2020.03.055

[6] Siegel RL, Miller KD, Jemal A. Cancer statistics, 2019. CA Cancer J Clin. 2019;69(1):7–34. 30620402 10.3322/caac.21551

[7] Alfred Witjes J, Lebret T, Compérat EM, Cowan NC, De Santis M, Bruins HM, et al. Updated 2016 EAU guidelines on muscle-invasive and metastatic bladder Cancer. Eur Urol. 2017;71(3):462–75. 27375033 10.1016/j.eururo.2016.06.020

[8] Clarke MF, Fuller M. Stem cells and cancer: two faces of eve. Cell. 2006;124(6):1111–5. 16564000 10.1016/j.cell.2006.03.011

[9] Visvader JE, Lindeman GJ. Cancer stem cells in solid tumours: accumulating evidence and unresolved questions. Nat Rev Cancer. 2008;8(10):755–68. 18784658 10.1038/nrc2499

[10] Hogarty MD, Liu X, Thompson PM, White PS, Sulman EP, Maris JM, et al. BIN1 inhibits colony formation and induces apoptosis in neuroblastoma cell lines with MYCN amplification. Med Pediatr Oncol. 2000;35(6):559–62. 11107117 10.1002/1096-911x(20001201)35:6<559::aid-mpo14>3.0.co;2-j

[11] Wechsler-Reya R, Elliott K, Herlyn M, Prendergast GC. The putative tumor suppressor BIN1 is a short-lived nuclear phosphoprotein, the localization of which is altered in malignant cells. Cancer Res. 1997;57(15):3258–63. 9242458

[12] Mao NC, Steingrimsson E, DuHadaway J, Wasserman W, Ruiz JC, Copeland NG, et al. The murine Bin1 gene functions early in myogenesis and defines a new region of synteny between mouse chromosome 18 and human chromosome 2. Genomics. 1999;56(1):51–8. 10036185 10.1006/geno.1998.5709

[13] DuHadaway JB, Sakamuro D, Ewert DL, Prendergast GC. Bin1 mediates apoptosis by c-Myc in transformed primary cells. Cancer Res. 2001;61(7):3151–6. 11306501

[14] Sakamuro D, Elliott KJ, Wechsler-Reya R, Prendergast GC. BIN1 is a novel MYC-interacting protein with features of a tumour suppressor. Nat Genet. 1996;14(1):69–77. 8782822 10.1038/ng0996-69

[15] Elliott K, Sakamuro D, Basu A, Du W, Wunner W, Staller P, et al. Bin1 functionally interacts with Myc and inhibits cell proliferation via multiple mechanisms. Oncogene. 1999;18(24):3564–73. 10380878 10.1038/sj.onc.1202670

[16] Elliott K, Ge K, Du W, Prendergast GC. The c-Myc-interacting adaptor protein Bin1 activates a caspase-independent cell death program. Oncogene. 2000;19(41):4669–84. 11032017 10.1038/sj.onc.1203681

[17] Galderisi U, Di Bernardo G, Cipollaro M, Jori FP, Piegari E, Cascino A, et al. Induction of apoptosis and differentiation in neuroblastoma and astrocytoma cells by the overexpression of Bin1, a novel myc interacting protein. J Cell Biochem. 1999;74(3):313–22. 10412034

[18] Ge K, DuHadaway J, Du W, Herlyn M, Rodeck U, Prendergast GC. Mechanism for elimination of a tumor suppressor: aberrant splicing of a brain-specific exon causes loss of function of Bin1 in melanoma. Proc Natl Acad Sci U S A. 1999;96(17):9689–94. 10449755 10.1073/pnas.96.17.9689 PMC22271

[19] Ge K, Duhadaway J, Sakamuro D, Wechsler-Reya R, Reynolds C, Prendergast GC. Losses of the tumor suppressor BIN1 in breast carcinoma are frequent and reflect deficits in programmed cell death capacity. Int J Cancer. 2000;85(3):376–83. 10652430

[20] Ge K, Minhas F, Duhadaway J, Mao NC, Wilson D, Buccafusca R, et al. Loss of heterozygosity and tumor suppressor activity of Bin1 in prostate carcinoma. Int J Cancer. 2000;86(2):155–61. 10738240 10.1002/(sici)1097-0215(20000415)86:2<155::aid-ijc2>3.0.co;2-m

[21] Vasiliou V, Nebert DW. Analysis and update of the human aldehyde dehydrogenase (ALDH) gene family. Hum Genomics. 2005;2(2):138–43. 16004729 10.1186/1479-7364-2-2-138 PMC3525259

[22] Maden M. Retinoid signalling in the development of the central nervous system. Nat Rev Neurosci. 2002;3(11):843–53. 12415292 10.1038/nrn963

[23] Gerber JM, Smith BD, Ngwang B, Zhang H, Vala MS, Morsberger L, et al. A clinically relevant population of leukemic CD34(+)CD38(-) cells in acute myeloid leukemia. Blood. 2012;119(15):3571–7. 22262762 10.1182/blood-2011-06-364182 PMC3325044

[24] Ginestier C, Hur MH, Charafe-Jauffret E, Monville F, Dutcher J, Brown M, et al. ALDH1 is a marker of normal and malignant human mammary stem cells and a predictor of poor clinical outcome. Cell Stem Cell. 2007;1(5):555–67. 18371393 10.1016/j.stem.2007.08.014 PMC2423808

[25] Storms RW, Trujillo AP, Springer JB, Shah L, Colvin OM, Ludeman SM, et al. Isolation of primitive human hematopoietic progenitors on the basis of aldehyde dehydrogenase activity. Proc Natl Acad Sci U S A. 1999;96(16):9118–23. 10430905 10.1073/pnas.96.16.9118 PMC17742

[26] Wan S, Cao J, Chen S, Yang J, Wang H, Wang C, et al. Construction of noninvasive prognostic model of bladder cancer patients based on urine proteomics and screening of natural compounds. J Cancer Res Clin Oncol. 2023;149(1):281–96. 36562811 10.1007/s00432-022-04524-x PMC11797276

[27] Zhang W, He X, Yin H, Cao W, Lin T, Chen W, et al. Allosteric activation of the metabolic enzyme GPD1 inhibits bladder cancer growth via the lysoPC-PAFR-TRPV2 axis. J Hematol Oncol. 2022;15(1):93. 35836291 10.1186/s13045-022-01312-5 PMC9284842

[28] Malta TM, Sokolov A, Gentles AJ, Burzykowski T, Poisson L, Weinstein JN, et al. Machine learning identifies stemness features Associated with Oncogenic Dedifferentiation. Cell. 2018;173(2):338–e354315. 29625051 10.1016/j.cell.2018.03.034 PMC5902191

[29] Langfelder P, Horvath S. WGCNA: an R package for weighted correlation network analysis. BMC Bioinformatics. 2008;9:559. 19114008 10.1186/1471-2105-9-559 PMC2631488

[30] Lenis AT, Lec PM, Chamie K, Mshs MD. Bladder Cancer: Rev JAMA. 2020;324(19):1980–91. 10.1001/jama.2020.17598 33201207

[31] Flores Monar GV, Reynolds T, Gordon M, Moon D, Moon C. Molecular markers for bladder Cancer screening: an insight into bladder Cancer and FDA-Approved biomarkers. Int J Mol Sci 2023, 24(18). 10.3390/ijms241814374 PMC10531979 37762677

[32] Kamat AM, Hahn NM, Efstathiou JA, Lerner SP, Malmstrom PU, Choi W, et al. Bladder cancer Lancet. 2016;388(10061):2796–810. 27345655 10.1016/S0140-6736(16)30512-8

[33] Springer SU, Chen CH, Rodriguez Pena MDC, Li L, Douville C, Wang Y et al. Non-invasive detection of urothelial cancer through the analysis of driver gene mutations and aneuploidy. Elife 2018, 7. 10.7554/eLife.32143 PMC5860864 29557778

[34] Dyrskjot L, Hansel DE, Efstathiou JA, Knowles MA, Galsky MD, Teoh J, et al. Bladder cancer. Nat Rev Dis Primers. 2023;9(1):58. 37884563 10.1038/s41572-023-00468-9 PMC11218610

[35] Tajiri T, Liu X, Thompson PM, Tanaka S, Suita S, Zhao H, et al. Expression of a MYCN-interacting isoform of the tumor suppressor BIN1 is reduced in neuroblastomas with unfavorable biological features. Clin Cancer Res. 2003;9(9):3345–55. 12960121

[36] Chang MY, Boulden J, Katz JB, Wang L, Meyer TJ, Soler AP, et al. Bin1 ablation increases susceptibility to cancer during aging, particularly lung cancer. Cancer Res. 2007;67(16):7605–12. 17699764 10.1158/0008-5472.CAN-07-1100

[37] Chang MY, Boulden J, Sutanto-Ward E, Duhadaway JB, Soler AP, Muller AJ, et al. Bin1 ablation in mammary gland delays tissue remodeling and drives cancer progression. Cancer Res. 2007;67(1):100–7. 17210688 10.1158/0008-5472.CAN-06-2742

[38] Li MX, Wang HY, Yuan CH, Ma ZL, Jiang B, Li L, et al. Establishment of a macrophage phenotypic switch related prognostic signature in patients with pancreatic Cancer. Front Oncol. 2021;11:619517. 33747931 10.3389/fonc.2021.619517 PMC7966706

[39] Ahmadzada T, Lee K, Clarke C, Cooper WA, Linton A, McCaughan B, et al. High BIN1 expression has a favorable prognosis in malignant pleural mesothelioma and is associated with tumor infiltrating lymphocytes. Lung Cancer. 2019;130:35–41. 30885349 10.1016/j.lungcan.2019.02.005

[40] Esmailzadeh S, Huang Y, Su MW, Zhou Y, Jiang X. BIN1 tumor suppressor regulates Fas/Fas ligand-mediated apoptosis through c-FLIP in cutaneous T-cell lymphoma. Leukemia. 2015;29(6):1402–13. 25578476 10.1038/leu.2015.9

[41] Prokic I, Cowling BS, Laporte J. Amphiphysin 2 (BIN1) in physiology and diseases. J Mol Med (Berl). 2014;92(5):453–63. 24590001 10.1007/s00109-014-1138-1

[42] Cassimere EK, Pyndiah S, Sakamuro D. The c-MYC-interacting proapoptotic tumor suppressor BIN1 is a transcriptional target for E2F1 in response to DNA damage. Cell Death Differ. 2009;16(12):1641–53. 19629135 10.1038/cdd.2009.98

[43] Mashima T. [Cancer Stem cells (CSCs) as a rational therapeutic Cancer target, and screening for CSC-targeting drugs]. Yakugaku Zasshi. 2017;137(2):129–32. 28154319 10.1248/yakushi.16-00229-1

[44] Tanabe S, Quader S, Cabral H, Ono R. Interplay of EMT and CSC in Cancer and the potential therapeutic strategies. Front Pharmacol. 2020;11:904. 32625096 10.3389/fphar.2020.00904 PMC7311659

[45] Roudi R, Korourian A, Shariftabrizi A, Madjd Z. Differential expression of Cancer Stem cell markers ALDH1 and CD133 in various Lung Cancer subtypes. Cancer Invest. 2015;33(7):294–302. 26046383 10.3109/07357907.2015.1034869

[46] Erfani E, Roudi R, Rakhshan A, Sabet MN, Shariftabrizi A, Madjd Z. Comparative expression analysis of putative cancer stem cell markers CD44 and ALDH1A1 in various skin cancer subtypes. Int J Biol Markers. 2016;31(1):e53–61. 26391478 10.5301/jbm.5000165

[47] Artavanis-Tsakonas S, Rand MD, Lake RJ. Notch signaling: cell fate control and signal integration in development. Science. 1999;284(5415):770–6. 10221902 10.1126/science.284.5415.770

[48] Rampias T, Vgenopoulou P, Avgeris M, Polyzos A, Stravodimos K, Valavanis C, et al. A new tumor suppressor role for the notch pathway in bladder cancer. Nat Med. 2014;20(10):1199–205. 25194568 10.1038/nm.3678

[49] Hayashi T, Gust KM, Wyatt AW, Goriki A, Jager W, Awrey S, et al. Not all NOTCH is created equal: the oncogenic role of NOTCH2 in bladder Cancer and its implications for targeted therapy. Clin Cancer Res. 2016;22(12):2981–92. 26769750 10.1158/1078-0432.CCR-15-2360

