# Supplementary material for: BIN1 inhibited tumor growth, metastasis and stemness by ALDH1/NOTCH pathway in bladder carcinoma
Source: Hereditas. 2025 Feb 27;162:29. doi: 10.1186/s41065-025-00384-w (PMC11866615; doi:10.1186/s41065-025-00384-w)
Supplement: Supplementary file 4 — Supplementary Material 4 [file 41065_2025_384_MOESM4_ESM.docx]

**Table S4. Primer sequences for qPCR.**

| Genes | Forward primer (5'-3') | Reverse primer (5'-3') |
| --- | --- | --- |
| BIN1 | CCCGACATCAAGTCACGCATT | TTTGAAGGGACTCGTAGTGGT |
| β-actin | CAGTCGGTTGGAGCGAGCAT | TGGCTTTTAGGATGGCAAGGGAC |
| Myc | GGCTCCTGGCAAAAGGTCA | CTGCGTAGTTGTGCTGATGT |
| ALDH1 | CCGTGGCGTACTATGGATGC | GCAGCAGACGATCTCTTTCGAT |
| OCT4 | TGAAGCTGGAGAAGGAGAAGCTG | TCTTTCTGCAGAGCTTTGATGTCCT |
| SOX2 | GCCGAGTGGAAACTTTTGTCG | GGCAGCGTGTACTTATCCTTCT |
| NANOG | TTTGTGGGCCTGAAGAAAACT | AGGGCTGTCCTGAATAAGCAG |
| EPCAM | AATCGTCAATGCCAGTGTACTT | TCTCATCGCAGTCAGGATCATAA |
