# Supplementary material for: BIN1 inhibited tumor growth, metastasis and stemness by ALDH1/NOTCH pathway in bladder carcinoma
Source: Hereditas. 2025 Feb 27;162:29. doi: 10.1186/s41065-025-00384-w (PMC11866615; doi:10.1186/s41065-025-00384-w)
Supplement: Supplementary file 5 — Supplementary Material 5 [file 41065_2025_384_MOESM5_ESM.docx]

**Table S5. Antibodies used for western blot.**

| Antibodies | Source | Cat. No. | Working  concentration |
| --- | --- | --- | --- |
| Mouse monoclonal anti-β-actin | Proteintech, China | 66009-1-Ig | 1:20000 |
| Rabbit polyclonal anti-BIN1 | Proteintech, China | 14647-1-AP | 1:2000 |
| Rabbit polyclonal anti-CYCLIN B | Affinity, USA | AF6168 | 1:1000 |
| Rabbit polyclonal anti-CYCLIN D | Proteintech, China | 26939-1-AP | 1:10000 |
| Rabbit polyclonal anti-CDK4 | Proteintech, China | 11026-1-AP | 1:1000 |
| Rabbit polyclonal anti-CDK1 | Proteintech, China | 19532-1-AP | 1:2000 |
| Rabbit polyclonal anti-N-cadherin | Proteintech, China | 22018-1-AP | 1:2000 |
| Rabbit polyclonal anti-E-cadherin | Proteintech, China | 20874-1-AP | 1:5000 |
| Rabbit polyclonal anti-Vimentin | Proteintech, China | 10366-1-AP | 1:2000 |
| Rabbit polyclonal anti-Myc | Proteintech, China | 10828-1-AP | 1:2000 |
| Mouse monoclonal anti-ALDH1 | Santa Cruz Biotechnology, USA | sc-374149 | 1:1000 |
| Mouse monoclonal anti-NANOG | Proteintech, China | 67255-1-Ig | 1:5000 |
| Mouse monoclonal anti-EPCAM | Santa Cruz Biotechnology, USA | sc-66020 | 1:1000 |
| Mouse monoclonal anti-KLF4 | Santa Cruz Biotechnology, USA | sc-166238 | 1:1000 |
| Rabbit polyclonal anti-OCT4 | Wanleibio, China | WL02020 | 1:1000 |
| Rabbit polyclonal anti-SOX2 | Wanleibio, China | WL03767 | 1:1000 |
| Rabbit monoclonal anti-NOTCH2 | Abcam, UK | ab307700 | 1:1000 |
| Rabbit polyclonal anti-TP53 | Affinity, USA | AF0879 | 1:1000 |
